# Changing Longitudinal Associations Between Home-Rearing Environments and Child Developmental Atypicality Across Two Decades

**DOI:** 10.3390/bs16081427

**Published:** 2026-08-19

**Authors:** Ying Huang, Lian Tong, Zhu Zhu, Yanlin Wang, Maiko Shigeeda, Xiang Li, Afsari Banu Alpona, Yuko Sawada, Akihiro Kakuda, Tokie Anme

**Affiliations:** 1 School of Comprehensive Human Science, University of Tsukuba, Tsukuba 305-8577, Japan; huangyvonne11@gmail.com (Y.H.);; 2 School of Public Health, Fudan University, Shanghai 200437, China; ltong@fudan.edu.cn; 3 School of Public Health and Nursing, Hangzhou Normal University, Hangzhou 311121, China; zhusunny1231@gmail.com; 4 College of Child Development and Education, Zhejiang Normal University, Hangzhou 311231, China; lixiangdufl@gmail.com; 5 Department of Physical Therapy, Morinomiya University of Medical Sciences, Osaka 559-8611, Japan; y-sawada@morinomiya-u.ac.jp (Y.S.);; 6 Faculty of Medicine, University of Tsukuba, Tsukuba 305-8575, Japan

**Keywords:** home-rearing environment, atypicality, child development, stimulation, social support

## Abstract

Home-rearing environments are integral to early childhood development. However, it remains unclear whether their developmental significance has changed over time. This study aimed to examine the temporal changes in home-rearing environments and their longitudinal associations with child developmental atypicality in Japan, using baseline assessments from 1999 to 2023 and follow-up assessments through 2024. Data were obtained from the Japanese Childcare Cohort Study, a nationwide childcare-based developmental surveillance project. The analysis included 40,627 1-year observation pairs of 18,210 children enrolled in childcare centers. Home-rearing environments were assessed using the Index of Child Care Environment, and developmental atypicality was evaluated using the Child Development Scale across six domains. Logistic regression models were used to examine the associations between baseline home-rearing environments and developmental atypicality 1 year later, adjusting for baseline developmental status, sex, and age. Generalized additive models and rolling-window analyses were used to assess temporal trends and time-varying associations. Overall, the Index of Child Care Environment scores increased gradually throughout the study period, although social stimulation declined after the mid-2010s. Higher-quality home-rearing environments were generally associated with a lower risk of subsequent developmental atypicality. Protective associations strengthened over time, particularly in the social, communication, vocabulary, and intellectual domains. Home-rearing environments and their developmental implications changed over the 25-year observation period. These findings highlight the continued importance of promoting direct caregiver–child interactions, including shared activities and social engagement. Early childhood surveillance should be periodically re-evaluated to remain relevant to evolving caregiving practices and family contexts.

## 1. Introduction

Early child development and caregiving environments are embedded within broader social contexts (Bronfenbrenner, 2000). Although associations between caregiving environments and child development are well established (Christensen et al., 2024), whether these associations remain stable over time has received considerably less attention. A long-term perspective may therefore provide insight into changes in child developmental patterns and home-rearing environments, as well as variation in their associations over time.

### 1.1. Changing Developmental Ecology and Early Childhood Development

Early childhood development is critical for lifelong health, education, and human capital formation (Schoellman, 2016; Victora et al., 2022). Over the past decades, societies worldwide have undergone rapid demographic, technological, and socioeconomic transformations that have altered the environments in which children grow and develop (OECD, 2025). Increasing digitalization (Brown & Council on Communications and Media, 2011; Mallawaarachchi et al., 2024), economic instability (Hamad & Rehkopf, 2016; Wickham et al., 2017), and public health crises (Sum et al., 2022) have reshaped the interaction ecology of children. Accumulating evidence suggests that developmental concerns among children remain substantial. Globally, developmental disabilities among children younger than 5 years continue to represent a major public health burden (Olusanya et al., 2023). In high-income countries, surveillance and survey data have documented increases in learning disabilities, developmental delays, and speech or language disorders among children between 2016 and 2021 (Leeb et al., 2024). Recent evidence from the COVID-19 pandemic further suggests that behavioral or conduct problems in children are related to rapid ecological disruptions (Lebrun-Harris et al., 2022).

Child developmental atypicality refers to deviations from age-expected developmental norms identified through structured screening or observational assessment, which are often detectable at earlier stages of life than formal clinical diagnoses (Lipkin et al., 2020). Evidence indicates that the early identification of developmental atypicality, followed by timely and appropriate support, can improve developmental trajectories, particularly in social communication, language, and adaptive functioning (Dawson et al., 2010). Therefore, developmental atypicality screening is valuable for tracking developmental patterns and identifying periods of vulnerability (Halfon et al., 2014).

### 1.2. Home-Rearing Environments

Beyond developmental screening, understanding early childhood development also requires consideration of the environments in which children grow and develop. According to the ecological systems theory, broader social conditions influence child development partly through proximal caregiving processes within children’s everyday environments (Bronfenbrenner, 2000). The Nurturing Care Framework identifies responsive caregiving, opportunities for early learning, safety and security, health, and nutrition as core conditions for healthy early development (World Health Organization, 2020). Among these, home-rearing environments encompass the organization of children’s everyday surroundings, including caregiver–child interaction, cognitive and social stimulation, and emotional and social support (Anme et al., 2013). A large body of evidence suggests that enriched and responsive home environments contribute to children’s cognitive functioning, including language, intelligence, and executive functioning (Jeong et al., 2021; Yang et al., 2021). Distinct home-rearing behaviors support development through domain-specific mechanisms. More stimulating home environments are associated with better receptive language and greater quality and complexity of expressive language (Lurie et al., 2021). Motor development more directly depends on opportunities for active play and reduced sedentary time (Zeng et al., 2017). Parenting intervention trials further suggest that strengthening responsive caregiving, parent–child interaction, and early learning opportunities improves cognitive, language, motor, socioemotional, and behavioral outcomes during the first 3 years of life (Jeong et al., 2021). Conversely, poor caregiving quality is associated with an increased risk of developmental delay (Wu et al., 2020).

### 1.3. Home Environments Are Historically and Socially Organized

The developmental relevance of home-rearing environments needs to be considered alongside their dynamic nature. Home-rearing environments evolve in response to broader societal changes (Merçon-Vargas et al., 2020), including labor participation, digital media use, and opportunities for interpersonal interaction (Braune-Krickau et al., 2021). Qualitative evidence supports this trend by showing that, compared with first-time parents in the 1970s, first-time parents in the 2010s perceived a substantially broader scope of parental responsibility, as government family life education, media, and commercial parenting services increasingly framed child well-being as the primary responsibility of parents (Lam et al., 2019). In England, comparisons of national cohorts in 1986 and 2006 revealed an increase in parental monitoring and expectations (Collishaw et al., 2012). Similarly, in a U.S. sample spanning approximately three decades, both cognitive stimulation and emotional support in the home environment improved among children up to the age of 10 years (O’Keefe & Rodgers, 2022).

Recent digitalization and pandemic-related disruptions further suggest that the composition and meaning of home-rearing environments may be changing (Brushe et al., 2024; Harverson et al., 2025; I. Takahashi et al., 2023). Studies from multiple countries have documented increased screen exposure, changed interaction patterns, and altered opportunities for social and language engagement during early childhood (Fish et al., 2026). In addition, parental technology use in the presence of a child, often described as “technoference,” has been associated with poorer cognitive and psychosocial outcomes and longer child screen time (Toledo-Vargas et al., 2025). Pandemic-era studies in Japan further suggest that rapid ecological disruption may simultaneously increase some family cohesion practices, such as shared meals and reduced punitive parenting, while reducing social support, social outings, and opportunities for interaction outside the home (Li et al., 2022). These findings suggest that home-rearing practices may vary across temporal and ecological contexts in both their composition and developmental significance.

### 1.4. Temporal Variation in Caregiving–Development Associations

Although ecological systems theory conceptualizes proximal caregiving processes as embedded within broader social contexts, relatively little is known about whether caregiving–development associations remain stable as these contexts change. Existing evidence has primarily focused on changes in the level and composition of home environments and parenting practices or on heterogeneity in caregiving effects across children’s age, socioeconomic conditions, and environmental adversity (Coffey & Snedeker, 2026; Schlesinger et al., 2025). If broad societal transformations reshape the developmental ecology of children, the developmental significance of caregiving environments may also evolve, rather than remain constant across generations. However, direct evidence on whether caregiving–development associations have changed over time remains scarce and is confined to adolescent samples. For example, based on US data collected between 1991 and 2019, a longitudinal study found that most adolescent-reported parenting practices remained broadly stable and that their protective associations with internalizing symptoms were largely consistent across decades (Kreski et al., 2023). Addressing this gap may improve our understanding of how rapidly changing societal conditions are associated with developmental processes and inform the design of future parenting support and early childhood intervention strategies.

The present study addresses these gaps using a 25-year repeated childcare-based developmental surveillance cohort in Japan. First, we examined the temporal changes in home-rearing environments across multiple caregiving domains. Second, we investigated the prospective associations between home-rearing environments and developmental atypicality 1 year later. Third, we examined whether caregiver-development associations varied across study periods and developmental domains. By integrating long-term developmental surveillance with repeated caregiving assessments, this study aimed to investigate whether the developmental significance of caregiving environments varied over time.

## 2. Materials and Methods

### 2.1. Study Design and Data Source

This study utilized data from the Japanese Childcare Cohort Study (CCC), an ongoing nationwide childcare-based developmental surveillance project conducted in government-authorized childcare centers in Japan since 1999 (Anme & Segal, 2007; Y. Takahashi et al., 2008). The CCC aims to monitor child development and caregiving environments in real-world childcare settings and to support improvements in childcare practices and family support systems. The present study used paired longitudinal data collected between 1999 and 2024, with baseline assessments conducted from 1999 to 2023 and corresponding 1-year follow-up assessments available through 2024.

### 2.2. Participants

Participating centers were eligible if they were officially authorized childcare centers in Japan and voluntarily participated in the annual developmental surveillance system during the study period. Over the 26-year study period, 112 government-authorized childcare centers provided data to the cohort.

The surveillance system targeted all children enrolled in the participating centers. For the present longitudinal analysis, annual assessments were linked across consecutive years using unique child identifiers. The final analytical dataset included 40,627 longitudinal observation pairs from 18,210 children, with repeated observations available for some children across multiple years. Written informed consent was obtained from parents or legal guardians prior to participation. Children with developmental disorders or disabilities were not excluded because the study aimed to capture developmental variations in inclusive real-world childcare settings in Japan (Zhu et al., 2021).

### 2.3. Assessment of Home-Rearing Environment

Children’s home-rearing environments were assessed using the Index of Child Care Environment (ICCE), a Japanese instrument developed to evaluate everyday caregiving experiences in family settings. The ICCE can be completed within approximately 20 min through caregiver self-report. Previous validation studies of the ICCE have demonstrated good psychometric properties, including a correlation coefficient of 0.76 with the Home Observation for Measurement of the Environment and a reproducibility coefficient of 0.91 (Anme et al., 2013).

The ICCE consists of 13 items across four domains. Human stimulation (five items) evaluates cognitively and emotionally engaging caregiver–child interactions, including playing with the child, reading books, singing songs, shared caregiving by spouses, and eating meals together as a family. Social stimulation (three items) assesses children’s opportunities for social and community engagement, including shopping, visiting parks, and interacting with friends or relatives with similarly aged children. Avoidance of restriction (two items) measures disciplinary approaches and caregiver responses to children’s behaviors, including reactions to children’s mistakes and the frequency of physical punishment. Social support (three items) evaluates the availability of caregiving assistance and emotional support, including support for childcare, consultation regarding parenting concerns, and communication with spouses about the child.

The items are assessed on a five-point scale: (1) rarely, (2) 1–2 times/month, (3) 1–2 times/week, (4) 3–4 times/week, and (5) almost every day. The responses were dichotomized according to the standardized ICCE scoring procedure. Specifically, “rarely” was coded as 0, whereas all other response categories were coded as 1, with higher scores indicating more favorable home-rearing environments. Two items comprising the Avoidance of restriction domain were reverse-coded. Specifically, for the item assessing responses to intentional misbehavior (e.g., a child spilling milk on purpose), punitive responses (e.g., hitting or scolding) were coded as 0, whereas preventive responses were coded as 1. For the item assessing physical punishment during the previous week, “never” was coded as 1, and any reported instance of hitting or kicking the child was coded as 0. The scores for each item were summed to generate the total and domain-specific ICCE scores (Anme et al., 2013).

Given the extended study period, we evaluated the temporal stability of the ICCE using ordinal alpha, based on tetrachoric correlations given the dichotomous and skewed nature of the items (Gadermann et al., 2014; Zumbo et al., 2007), and multigroup confirmatory factor analysis (MGCFA) (Sigerson et al., 2017; Svetina et al., 2020). Baseline years were divided into two periods, before 2010 and 2010 onward, to create broadly comparable samples for assessing temporal stability. Internal consistency was acceptable overall (ordinal α = 0.800) and broadly comparable before and after 2010 (Table S1). Measurement invariance across periods was examined using MGCFA for the dichotomous ICCE items. Under the delta parameterization, we compared a configural model with a more constrained model in which factor loadings were set equal across periods. The loading-constrained model showed no meaningful deterioration in model fit compared with the configural model (ΔCFI = 0.007, ΔRMSEA = −0.003, and ΔSRMR = 0.004; Table S2), indicating broadly stable factor-loading patterns over time (Chen, 2007). Absolute model fit was moderate (configural CFI = 0.863, RMSEA = 0.047, SRMR = 0.112), which may partly reflect the two-indicator Avoidance restriction factor and a high latent correlation between the Human stimulation and Support factors. As these factors nonetheless correspond to conceptually and behaviorally distinct caregiving items, the original four-factor structure was retained for all primary analyses.

### 2.4. Assessment of Child Development

Child development was assessed annually using the Child Development Scale (CDS), a standardized screening instrument widely used in Japanese childcare settings (Anme et al., 2007). The CDS has been normed in Japan, and it identifies approximately 10% of children as performing below an age-appropriate developmental level (Anme & Segal, 2004). The test–retest reliability coefficients for the six developmental domains range from 82.5% to 97.9% (Anme et al., 2007).

The CDS assesses six developmental domains: (1) gross motor skills, (2) fine motor skills, (3) social competence, (4) communication skills, (5) vocabulary, and (6) intellectual functioning. These domains can be conceptually grouped into three broader developmental dimensions: motor development (gross and fine motor skills), socioemotional development (social competence and communication skills), and language–cognitive development (vocabulary and intellectual functioning). The scale comprises 33 developmental levels ranging from 0 to 90 months. Developmental status is determined by comparing each child’s assessed developmental level with age-expected norms.

All participating childcare professionals received at least 8 h of standardized training prior to assessment administration, with agreement rates exceeding 85%. Childcare professionals evaluated the appropriate test level for each child based on daily behavioral observations. Children were classified as having developmental atypicality when their assessed developmental levels were more than three levels below the expected level for their chronological age (Anme et al., 2007). Developmental classifications were derived using automated calculations implemented in Microsoft Excel Visual Basic for Applications.

Annual prevalence was calculated as the proportion of children classified as having atypical development among those with valid, non-missing assessments for each study year and developmental domain. Children with missing data for a given developmental domain were excluded from the denominators of domain-specific prevalence calculations.

### 2.5. Longitudinal Paired Dataset Construction

One-year longitudinal paired datasets were generated by linking consecutive annual assessments of the same child. Each observation pair comprised baseline measures at year t and follow-up developmental outcomes at year t + 1. Baseline ICCE dimensions, baseline CDS-based atypicality, sex, and age category at year t were linked to CDS-based atypicality outcomes at year t + 1. Binary CDS-based atypicality variables were generated for each developmental domain. Invalid entries, including non-numeric responses and error codes, were treated as missing values. Children with missing outcome, exposure, sex, or age data were excluded from the corresponding analyses.

### 2.6. Rolling-Window and Time-Varying Association Analyses

To examine temporal variation in the associations between ICCE dimensions and subsequent developmental atypicality, rolling-window logistic regression analyses were conducted using overlapping 3-year windows across the study period. The rolling-window approach was used to reduce instability arising from annual fluctuations in sample size while preserving temporal sensitivity to changing associations over time (Ben Amor et al., 2016; Zivot & Wang, 2003). Each rolling estimate was assigned to the midpoint year of the corresponding 3-year window. For each center year, data from the center year and its two adjacent years were combined. For example, the rolling OR for 2010 was estimated using data from 2009, 2010, and 2011. For each window, logistic regression models were fitted separately for each ICCE dimension and CDS developmental domain. The dependent variable was CDS-based atypicality at year t + 1, and the independent variables were baseline ICCE score, baseline CDS-based atypicality, sex, and age category. Odds ratios (ORs) and 95% confidence intervals (CIs) for the ICCE variables were estimated for each rolling period.

### 2.7. Statistical Analysis

The statistical analyses were conducted in four sequential steps. First, temporal trends in caregiving environments were examined using generalized additive models (GAMs). Second, overall longitudinal associations of caregiving environments on subsequent developmental atypicality were estimated using logistic regression models. Third, time interaction models were fitted to assess whether these longitudinal associations changed across the study period. Finally, rolling-window logistic regression and GAM smoothing were used to characterize the temporal trajectories of the estimated associations.

Temporal trends in ICCE dimensions across baseline years 1999 to 2023 were examined using generalized additive models (GAMs) with smooth functions of calendar year. Smoothing parameters were estimated using a restricted maximum likelihood (REML); effective degrees of freedom (edf) were used to characterize the complexity of nonlinear temporal trajectories (Wood, 2011).

Longitudinal associations between baseline ICCE dimensions and CDS-based atypicality 1 year later were examined using logistic regression models. Separate models were fitted to each ICCE dimension and CDS developmental domain. The dependent variable was CDS-based atypicality at year t + 1, whereas independent variables included the baseline ICCE dimension at year t, baseline CDS-based atypicality, sex, and age category. ORs and 95% CIs were estimated. To examine whether ICCE–development associations varied across calendar years, interaction models including ICCE × calendar year interaction terms were fitted. The calendar year was centered on 2010 to improve the interpretability of the main effects (Oshio, 2020).

To further characterize temporal changes in the estimated associations of caregiving environments, rolling-window logistic regression analyses were performed using overlapping 3-year windows across the study period (Ben Amor et al., 2016; Zivot & Wang, 2003). GAMs were then fitted to the rolling log OR estimates using penalized regression splines (Cadarso-Suárez et al., 2005). The fitted values and corresponding 95% confidence intervals were exponentiated and presented as smoothed OR trajectories to visualize temporal changes in the associations of caregiving environments across the study period. To account for multiple testing, the Benjamini–Hochberg false discovery rate (FDR) procedure was applied separately to the main-effect and calendar-year interaction analyses (Benjamini & Hochberg, 1995). Given the exploratory nature of the analyses and the correlation among related developmental outcomes, FDR-adjusted results were used for interpretation (Glickman et al., 2014).

Sensitivity analyses using cluster-robust standard errors were conducted for both the main models and interaction models between ICCE dimensions and calendar year to account for within-child and within-childcare-center dependence (Huang et al., 2023). Robust standard errors were estimated separately at the child and childcare-center levels, and 95% CIs and *p*-values were recalculated. The sensitivity analyses otherwise retained the same model specifications and covariate adjustments as the corresponding primary analyses. All statistical analyses were conducted using R version 4.5.0.

### 2.8. Ethical Considerations

The study was conducted in accordance with the Declaration of Helsinki and approved by the Medical Ethics Committee of the Faculty of Medicine, University of Tsukuba, Japan (Approval No. 1657-2; updated in November 2024). This study was conducted in accordance with the National Ethical Guidelines for Epidemiological Research of Japan. All data were anonymized prior to the analysis. Written informed consent was obtained from parents or legal guardians prior to participation.

## 3. Results

### 3.1. Sample Characteristics

The longitudinal dataset included 40,627 observation pairs from 18,210 children. Boys accounted for 52.15% of the participants. The mean age increased from 41.54 months at baseline to 53.15 months at follow-up. At baseline, 56.7% of the observations were from children aged 0–3 years; 63.7% of the follow-up observations were from children aged 4–6 years. Mean ICCE scores were generally stable between baseline and follow-up. Across developmental domains, vocabulary atypicality showed the highest prevalence at both baseline (6.9%, 95% CI 6.6–7.1) and follow-up (5.3%, 95% CI 5.1–5.6), whereas gross motor atypicality showed the lowest prevalence (2.5%, 95% CI 2.4–2.7 at baseline; 2.0%, 95% CI 1.8–2.1 at follow-up). The baseline and follow-up demographic and developmental characteristics of the longitudinal observation pairs are presented in Table 1. Annual demographic characteristics of the study population, annual mean ICCE scores, and the prevalence of child developmental atypicality are provided in Tables S3–S5. The annual numbers of valid observations for the ICCE and CDS variables are presented in Tables S6 and S7.

### 3.2. Trends in the Home-Rearing Environment

**Figure 1:**
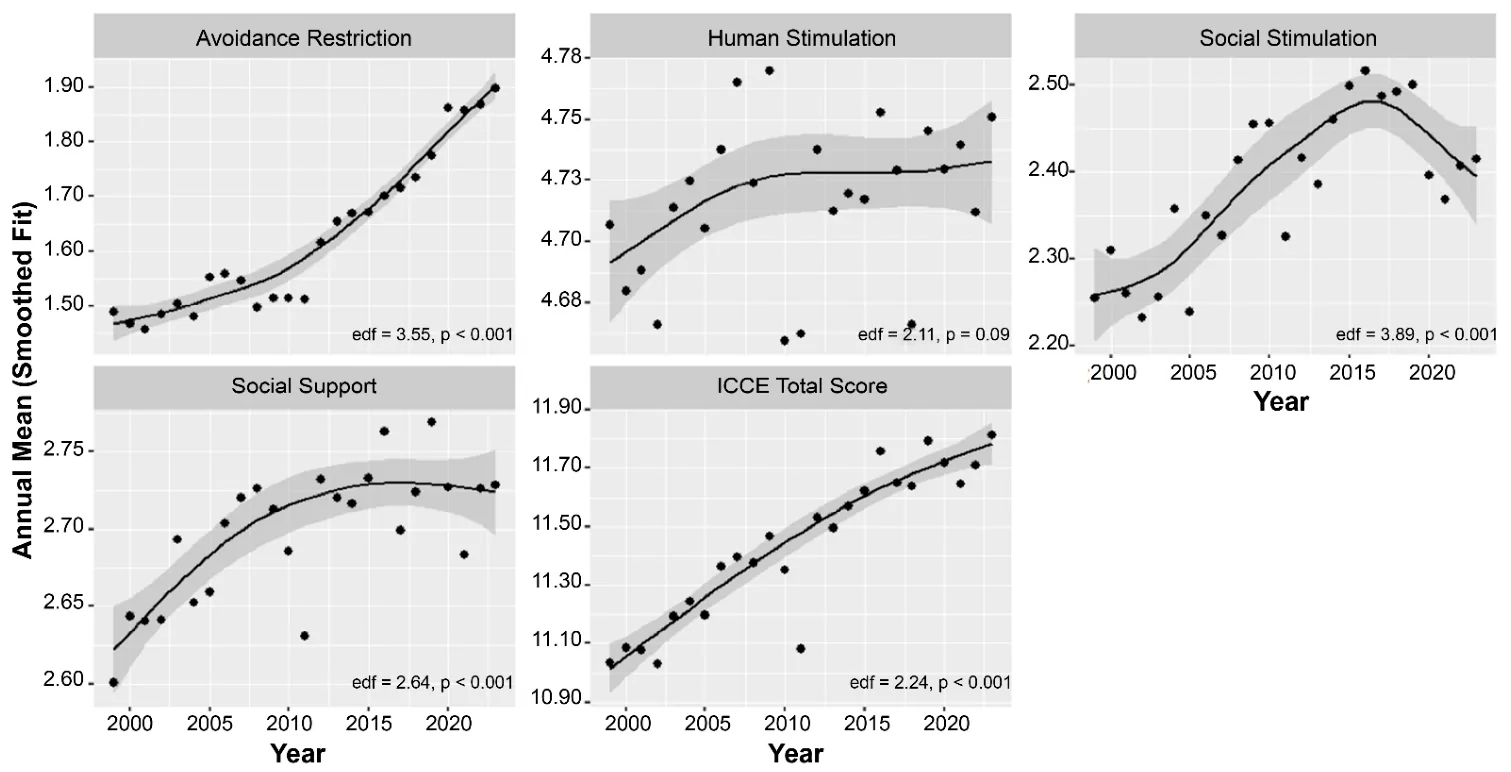
GAM-smoothed annual ICCE mean values with 95% confidence intervals. Note: generalized additive models (GAMs); ICCE, Index of Child Care Environment. Higher effective degrees of freedom (edf) indicate more complex nonlinear temporal trajectories. Solid lines represent the GAM-smoothed estimates of annual mean ICCE scores, with shaded areas indicating 95% confidence intervals. Points represent the observed annual mean ICCE scores.

Overall, the ICCE total score, reflecting the overall quality of the family caregiving environment, increased gradually across the study period from 1999 to 2023. GAM smoothing demonstrated a significant nonlinear upward trajectory (edf = 2.24, *p* < 0.001). Among the caregiving dimensions, opportunities for social stimulation exhibited the most pronounced nonlinear pattern, increasing from 1999 to 2016 and then declining slightly from 2016 to 2023 (edf = 3.89, *p* < 0.001). Avoidance of restrictive parenting practices increased steadily throughout the study period, whereas human stimulation activities and perceived social support remained relatively stable. Figure 1 illustrates the annual mean ICCE scores with GAM-smoothed trajectories, and the corresponding annual mean ICCE scores are presented in Table S4.

### 3.3. Overall Associations of the Caregiving Environment with Developmental Atypicality

**Figure 2:**
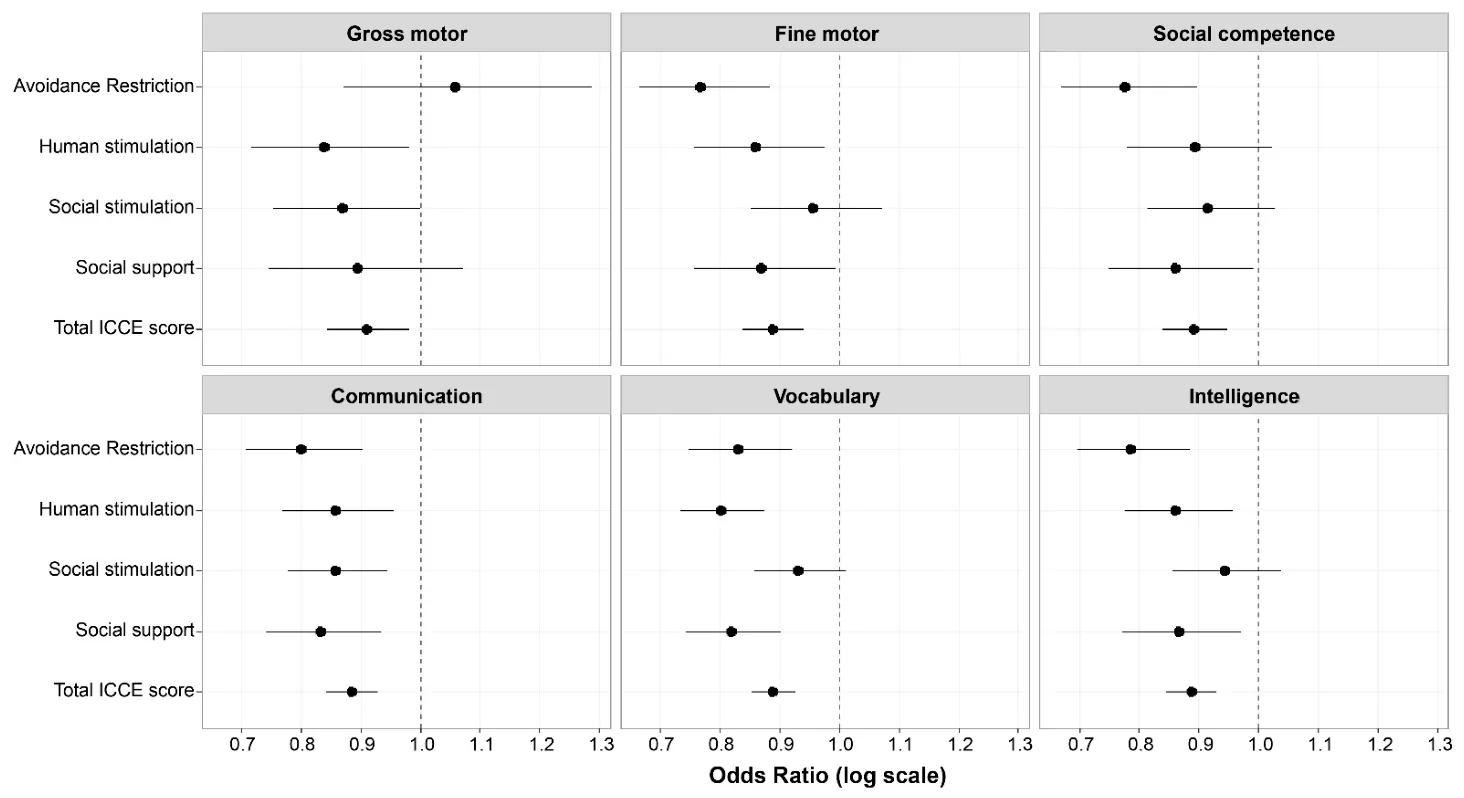
Associations between ICCE dimensions at year t and CDS alert status at year t + 1. Note. ICCE, Index of Child Care Environment. All models were adjusted for the baseline CDS alert status (year t), sex, and age.

After adjusting for baseline child atypicality, sex, and age, more positive caregiving environments, as measured by the ICCE, were generally associated with reduced likelihood of subsequent developmental atypicality across developmental domains (Table 2 and Figure 2). Higher overall ICCE scores were consistently associated with lower odds of atypicality in all six developmental domains (ORs = 0.89–0.91, all *p* < 0.05). For motor development, human stimulation activities demonstrated protective associations on both gross motor (OR = 0.84, *p* = 0.028) and fine motor development (OR = 0.86, *p* = 0.019), reflected by reduced likelihoods of subsequent atypicality. Similarly, avoidance of restrictive parenting practices was associated with a lower likelihood of fine motor developmental atypicality (OR = 0.77, *p* < 0.001). After FDR correction, 20 of the 22 nominally significant main associations remained significant. The associations of social support with fine motor atypicality (*p* = 0.043, FDR-adjusted *p* = 0.058) and social competence atypicality (*p* = 0.039, FDR-adjusted *p* = 0.056) no longer reached statistical significance.

### 3.4. Time-Varying Associations of the Caregiving Environment

To examine whether the associations of the caregiving environment varied over time, interaction terms between each ICCE dimension and calendar year were introduced into the models. Interaction ORs indicate the strength of the association between ICCE scores and subsequent developmental atypicality changed over time. In the present study, because the overall associations of ICCE were consistently protective (ORs < 1), interaction ORs below 1 indicate progressively stronger protective associations over time (Table 3).

For motor development, progressively stronger protective associations were observed for the overall caregiving environment on both gross motor (interaction OR = 0.984, *p* = 0.003) and fine motor development (interaction OR = 0.990, *p* = 0.012). Avoidance of restrictive parenting practices and social support did not show significant interactions with calendar year.

The total caregiving environment was found to have increasingly strong protective associations with lower odds of social competence atypicality (interaction OR = 0.988, *p* = 0.008) and communication development (interaction OR = 0.987, *p* < 0.001). Significant temporal strengthening was also observed for human stimulation activities and opportunities for social stimulation on social-emotional development.

For language development, the protective associations of the overall caregiving environment strengthened over time for both vocabulary (interaction OR = 0.987, *p* < 0.001) and intelligence development (interaction OR = 0.983, *p* < 0.001). Significant temporal strengthening was also observed for human stimulation activities and opportunities for social stimulation on both vocabulary and intelligence. Social support showed statistically significant interactions with calendar year for vocabulary (interaction OR = 0.986, *p* = 0.031) and intelligence development (interaction OR = 0.981, *p* = 0.015, Table 3). After FDR correction, all 20 nominally significant calendar-year interaction terms remained statistically significant.

### 3.5. Trends of the Associations of the Home-Rearing Environment

**Figure 3:**
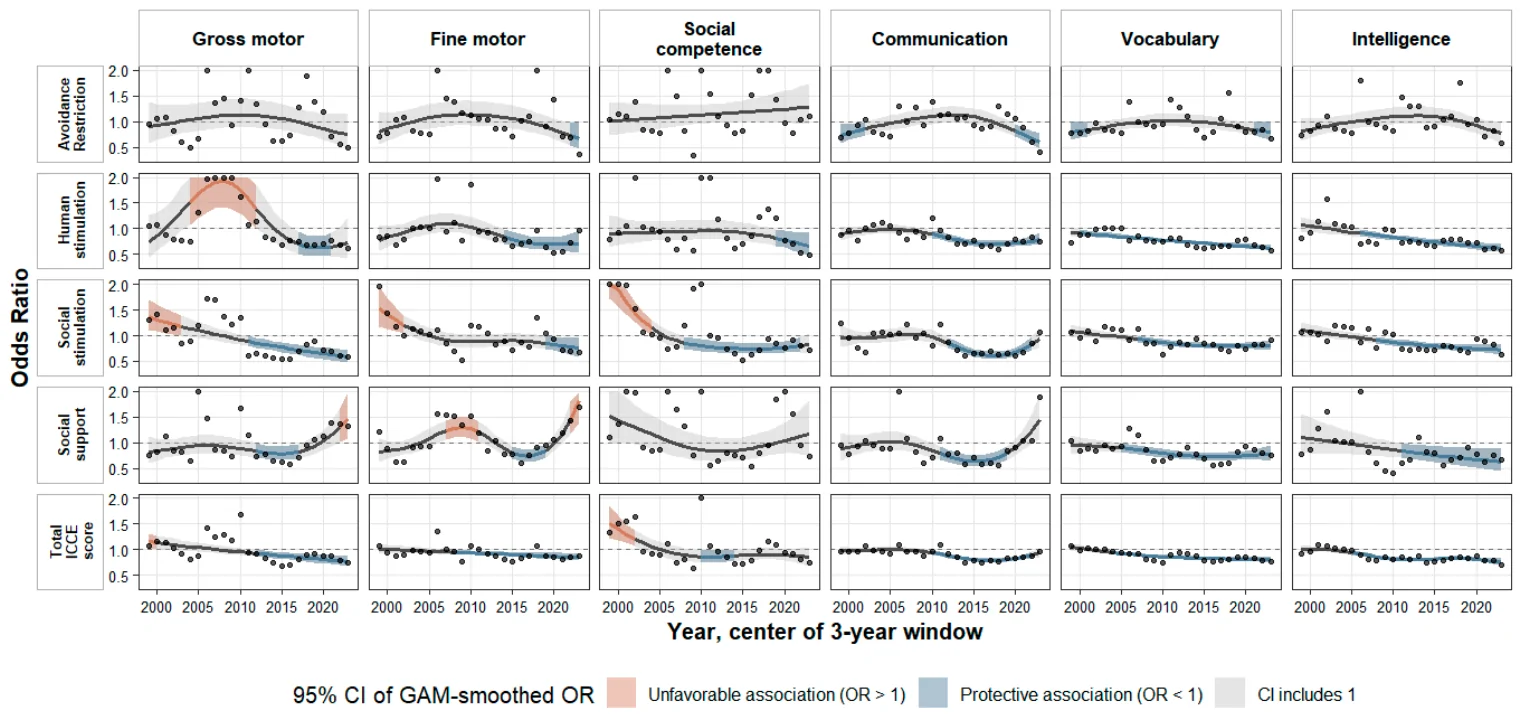
Three-year rolling window estimates of the associations between ICCE dimensions and child atypicality at year t + 1. Note. ICCE, Index of Child Care Environment. Black points represent individual three-year rolling-window OR estimates. The black line and shaded band represent the trajectory and 95% CI obtained by fitting a generalized additive model (GAM). For visual clarity, estimates exceeding 2.0 are displayed truncated at the upper axis boundary.

The GAMs were applied to the rolling three-year log(OR) estimates to characterize and visualize the overall temporal trends in these associations (Table 4 and Figure 3). Higher estimated degrees of freedom (edf) indicated more complex nonlinear temporal variations, whereas edf values close to 1 suggested relatively linear trajectories over time.

For motor development, several ICCE dimensions showed temporal variation in their estimated associations. Significant nonlinear trajectories were identified for human stimulation activities in both gross (edf = 3.24, *p* = 0.002) and fine motor development (edf = 2.88, *p* = 0.021), as well as for opportunities for social stimulation and social support in fine motor development (Table 4). Linear strengthening trajectories were observed for the overall caregiving environment in both gross motor and fine motor development (edf = 1.00, *p* < 0.05). Social support showed a nonlinear trajectory in relation to fine motor atypicality (edf = 3.77, *p* < 0.001; Figure 3). The smoothed trajectory suggested possible changes over time, although the apparent shift toward a more positive association in recent years should be interpreted cautiously.

For social-emotional development, social stimulation showed temporal variation in its association with social competence atypicality (edf = 2.99, *p* < 0.001). Communication development further exhibited significant nonlinear trajectories for avoidance of restrictive parenting practices (edf = 2.89, *p* = 0.005), human stimulation activities (edf = 3.25, *p* < 0.001), and social support (edf = 3.44, *p* = 0.003) (Figure 3).

Cognitive-language development exhibited the most consistent temporal trajectories among all developmental domains. Human stimulation activities demonstrated approximately linear strengthening protective associations with both vocabulary and intelligence development (edf = 1.00, *p* < 0.001). The GAM-smoothed trajectories for vocabulary and intelligence development demonstrated progressively stronger protective associations over time (Figure 3). Individual rolling-window estimates are provided in Supplementary File S2.

### 3.6. Sensitivity Analyses

Sensitivity analyses accounting for repeated measurements within children and clustering of children within childcare centers using cluster-robust standard errors showed that the main findings were generally robust (Tables S8 and S9). Of the associations that were statistically significant in the primary analyses, only the association between total ICCE score and gross motor atypicality became non-significant when standard errors were clustered by childcare center (*p* = 0.058), while the association between social support and fine motor atypicality became non-significant when clustered by child (*p* = 0.061). Similarly, most interactions between ICCE dimensions and calendar year remained significant after clustering adjustment. The interaction between human stimulation and calendar year for gross motor atypicality became non-significant when clustering by childcare center (*p* = 0.074). Overall, accounting for within-cluster dependence altered the precision of some estimates but did not materially change the overall pattern of findings.

## 4. Discussion

### 4.1. Principal Findings

The present study examined temporal trends in home-rearing environments and their time-varying associations with developmental atypicality 1 year later using a cohort spanning 25 years in Japan. Our study found that (a) overall home-rearing environments and most subdomains improved across the study period, although Social stimulation showed a decline from the mid-to-late 2010s onward; (b) higher-quality home-rearing environments were associated with lower risks of developmental atypicality 1 year later across developmental domains across the overall study period; and (c) the protective associations between home-rearing environments and developmental outcomes strengthened, particularly in social and language developmental domains.

### 4.2. Declining Social Stimulation

Social stimulation showed a declining trend from the mid-to-late 2010s onward. This domain highlights children’s opportunities for face-to-face and community-based social activities, including visiting parks, shopping with caregivers, and engaging with classmates or relatives of the same age. This decline suggests that these forms of social engagement have become less frequent in recent years, although the present data cannot establish the specific factors driving this change. One plausible contributing mechanism is the rapid diffusion of smartphones and mobile social media use in Japan, which broadly coincided with the observed decline. Since the mid-2010s, smartphones, tablets, and algorithm-driven short video platforms have been rapidly integrated into everyday family life (Canadian Paediatric Society et al., 2017; Mann et al., 2025). From 8.0% in 2010 to nearly 91.1% in 2015 for females aged 20–34, and from 14.3% in 2010 to 87.2% in 2015 for males aged 20–34, smartphone ownership among young adults in Japan has grown dramatically (Dentsu, 2015). Prior research suggests that increasing parental smartphone and short-video use reduces opportunities for caregiver–child interaction and shared social activities (Braune-Krickau et al., 2021; Luef, 2018). The implications of digitalization extend beyond parental smartphone use, as digital media exposure among children has also recently increased. Nearly half of children watched short-form videos on platforms, with 16% engaging daily in such activities (Mann et al., 2025). Early screen exposure was associated with lower engagement in shared reading activities, reduced outdoor play frequency, and fewer face-to-face social interactions (McArthur et al., 2021; Sugiyama et al., 2023). In summary, these parallel trends suggest digitalization as one plausible mechanism, though this interpretation remains speculative given the lack of direct measurement in this study. Continued monitoring of this mechanism is warranted in future work.

### 4.3. Strengthening Associations of Stimulation-Related ICCE Dimensions

The present findings further suggest that the prospective associations between stimulation-related caregiving behavior and child language and socioemotional development have been strengthened recently. Human stimulation activities include playing, reading, singing, shared caregiving, and family mealtime interactions, whereas social stimulation captures children’s opportunities for peer interaction and community-based social engagement. Recent OECD evidence similarly highlights the importance of parent–child activities for children’s early learning and development (OECD, 2026). As discussed above, growing evidence suggests that contemporary childhood environments have increasingly shifted toward greater digital media exposure, reduced outdoor play, and fewer face-to-face social interactions (Brown & Council on Communications and Media, 2011; Mallawaarachchi et al., 2024). The resource substitution theory proposes that when one developmental or social resource becomes less available, the influence of alternative resources may become increasingly amplified (Andersson, 2016; Conti & Heckman, 2010). Consistent with this perspective, recent guidance recommends replacing screen-based activities with developmentally meaningful alternatives, such as child-centered play and interaction (Munzer et al., 2026). Accordingly, a reduction in interpersonal and socially interactive experiences increases the developmental dependence of children on stimulation-related caregiving environments within families (Settels, 2022). As opportunities for socially embedded interactions become increasingly limited, stimulation-related experiences within families may function as critical developmental inputs and may show stronger associations with developmental outcomes.

Notably, the clearest temporal strengthening of associations was observed in language outcomes. These domain-specific patterns are consistent with social interactionist theories suggesting that language, communication, and cognitive functions are particularly dependent on reciprocal caregiver–child interactions and socially embedded stimulation during early childhood (Ghani et al., 2022). Furthermore, previous reviews have consistently shown that the strongest intervention effects are on language and cognitive outcomes, followed by socioemotional development, whereas the effects on motor development are generally smaller (Britto et al., 2017; Jeong et al., 2021). These findings suggest that socially and cognitively stimulating caregiver–child interactions may be particularly relevant for children growing up in environments with fewer opportunities for interpersonal interaction. Despite this consistency with previous evidence, it should be noted that the associations observed in the present study were generally modest in magnitude. Nevertheless, as stimulation-related caregiving behaviors represent repeatedly experienced aspects of children’s everyday environments, even modest associations may have relevance from a population-level perspective.

### 4.4. Temporal Variation

The smoothed trajectory of the association between social support and fine motor development suggested a possible shift toward a positive association with developmental atypicality in recent years. Although this pattern was not fully stable in the sensitivity analyses and should therefore be interpreted cautiously, it warrants continued attention as a potential indicator of changing family support needs. The social support dimension includes support with childcare, consultation regarding parenting concerns, and communication with spouses regarding children. From a family systems perspective, support-seeking and caregiving responses are embedded within changing family needs and caregiving conditions (Alonso & Little, 2019; Sawrikar et al., 2026). One possible explanation is that increased caregiver stress and family mental health concerns may have further accelerated support-seeking behaviors among families raising children with developmental or psychosocial difficulties (Racine et al., 2022). Another possibility is that recent family-support and early-intervention policies in Japan have increasingly emphasized early identification, parenting consultation, and community-based child-rearing support, potentially increasing accessibility of support services and encouraging families to seek assistance at earlier stages (Children and Families Agency, 2019; Government of Japan, 2012; The Japan Ministry of Health, Labour and Welfare, 2004). Changes observed around and after 2020 should also be considered in the context of the COVID-19 pandemic. Therefore, some of the recent short-term variation may reflect pandemic-related disruptions in addition to longer-term temporal changes (Alonso & Little, 2019; Buck et al., 2022; Racine et al., 2022). However, relationships between developmental concerns and subsequent support-seeking were not explicitly modeled in the present study. Further studies using longitudinal methods designed to examine reciprocal associations are needed to clarify the direction and underlying mechanisms.

### 4.5. Policy and Public Health Implications

Stimulation-related caregiving experiences may become increasingly important in childhood environments characterized by reduced face-to-face interactions and greater digital engagement. Therefore, policies and interventions should continue to promote caregiver–child interactions, peer engagement, outdoor play, and community-based social experiences during early childhood. In addition, support-seeking patterns warrant continued monitoring to better understand changing family and developmental needs over time.

### 4.6. Limitations and Future Directions

This study had several limitations that should be considered when interpreting the findings. First, the participating childcare centers were enrolled voluntarily and were not nationally representative, which may limit the generalizability of the findings. Second, ICCE assessments were based on caregiver-reported questionnaires and may therefore be influenced by respondents’ psychological states. Parental mental health was not available and could not be adjusted for. Although developmental outcomes were independently assessed by childcare professionals, reporting bias in the ICCE and residual confounding by caregiver psychological characteristics cannot be excluded. Third, this study did not directly examine the specific mechanisms underlying observed changes over time, such as exposure to digital media. Relatedly, because the ICCE was developed to capture relatively traditional forms of caregiver-child interaction, it may not fully reflect newer forms of interaction that have emerged over the study period, including digitally mediated caregiving practices. Future work should consider updating or supplementing the instrument to capture such changes. Fourth, family socioeconomic characteristics, including parental education and occupation, could not be adequately adjusted for because of missing data for these variables. Given recent evidence highlighting the importance of socioeconomic conditions for child developmental outcomes (Marek et al., 2026), residual confounding by socioeconomic and demographic factors cannot be excluded. Fifth, temporal changes in developmental awareness, childcare policies, and social contexts across the 25-year study period may have influenced caregiving behaviors and reporting practices. The implementation of developmental disability support policies in Japan around 2005 may have increased awareness and attention toward developmental difficulties, potentially contributing to detection or reporting shifts during certain periods (The Japan Ministry of Health, Labour and Welfare, 2004). Finally, some temporal fluctuations in the rolling-window and year-specific analyses may partly reflect the statistical instability related to varying annual sample sizes across developmental domains. Future studies should explore the specific mechanisms underlying these temporal changes by incorporating direct measures of potential contextual factors.

## 5. Conclusions

This study demonstrates that home-rearing environments and their developmental associations have changed over the 25-year observation period. Stimulation-related caregiving environments have shown increasing protective associations with children’s language and socioemotional development over time, despite declining levels of social stimulation in recent years. Theoretically, these findings suggest that the developmental relevance of caregiving experiences may be relational rather than fixed, varying across temporal contexts. These findings highlight the continued importance of promoting direct caregiver–child interactions. Future research and developmental surveillance should also consider emerging forms of caregiver–child interaction that may not be fully captured by traditional measures.

## Figures and Tables

**Table 1:** Baseline and follow-up characteristics of the longitudinal observation pairs.

Category	Baseline	Follow-Up
Sample size		
Observation pairs	40,627	
Unique children	18,210	
Median observation pairs per child	2 (IQR 1–3)	
Sex, *n* (%)		
Girls	19,340 (47.8)	
Boys	21,100 (52.2)	
Age months, mean (SD)	41.54 (16.76)	53.15 (16.85)
Age group, *n* (%)		
0–3 years	22,220 (56.7)	14,188 (36.3)
4–6 years	16,938 (43.3)	24,951 (63.7)
ICCE score, mean (SD)		
Avoidance restriction	1.62 (0.55)	1.61 (0.55)
Human stimulation	4.72 (0.61)	4.69 (0.64)
Social stimulation	2.37 (0.71)	2.40 (0.69)
Social support	2.70 (0.58)	2.70 (0.57)
Total ICCE score	11.45 (1.44)	11.44 (1.46)
CDS-based atypicality, (%, 95% CI)		
Gross motor	2.5, 2.4–2.7	2.0, 1.8–2.1
Fine motor	3.1, 2.9–3.3	2.7, 2.5–2.8
Social competence	3.2, 3.0–3.3	2.5, 2.3–2.6
Communication	4.3, 4.1–4.5	3.8, 3.6–4.0
Vocabulary	6.9, 6.6–7.1	5.3, 5.1–5.6
Intelligence	3.7, 3.6–3.9	3.7, 3.5–3.9

Note: SD, standard deviation; CI, confidence interval; ICCE, Index of Child Care Environment; CDS, Child Development Scale.

**Table 2:** Longitudinal associations between baseline ICCE dimensions and subsequent CDS-based atypicality.

Outcome	ICCE Dimension	OR (95% CI)	*p*-Value	FDR-Adjusted *p*
Gross motor	Avoidance restriction	1.06 (0.87–1.29)	0.566	0.566
	Human stimulation	0.84 (0.72–0.98)	0.028	0.042
	Social stimulation	0.87 (0.75–1.00)	0.052	0.068
	Social support	0.89 (0.75–1.07)	0.230	0.248
	Total ICCE score	0.91 (0.84–0.98)	0.015	0.024
Fine motor	Avoidance restriction	0.77 (0.66–0.88)	<0.001	<0.001
	Human stimulation	0.86 (0.76–0.98)	0.019	0.030
	Social stimulation	0.96 (0.85–1.07)	0.434	0.449
	Social support	0.87 (0.76–1.00)	0.043	0.058
	Total ICCE score	0.89 (0.84–0.94)	<0.001	<0.001
Social competence	Avoidance restriction	0.78 (0.67–0.90)	<0.001	0.002
	Human stimulation	0.89 (0.78–1.02)	0.102	0.122
	Social stimulation	0.92 (0.81–1.03)	0.140	0.161
	Social support	0.86 (0.75–0.99)	0.039	0.056
	Total ICCE score	0.89 (0.84–0.95)	<0.001	<0.001
Communication	Avoidance restriction	0.80 (0.71–0.90)	<0.001	<0.001
	Human stimulation	0.86 (0.77–0.96)	0.006	0.011
	Social stimulation	0.86 (0.78–0.94)	0.002	0.004
	Social support	0.83 (0.74–0.94)	0.002	0.004
	Total ICCE score	0.89 (0.84–0.93)	<0.001	<0.001
Vocabulary	Avoidance restriction	0.83 (0.75–0.92)	<0.001	0.001
	Human stimulation	0.80 (0.73–0.88)	<0.001	<0.001
	Social stimulation	0.93 (0.86–1.01)	0.090	0.113
	Social support	0.82 (0.74–0.90)	<0.001	<0.001
	Total ICCE score	0.89 (0.85–0.93)	<0.001	<0.001
Intelligence	Avoidance restriction	0.79 (0.70–0.89)	<0.001	<0.001
	Human stimulation	0.86 (0.78–0.96)	0.006	0.011
	Social stimulation	0.94 (0.86–1.04)	0.232	0.248
	Social support	0.87 (0.77–0.97)	0.014	0.024
	Total ICCE score	0.89 (0.85–0.93)	<0.001	<0.001

Note: ICCE, Index of Child Care Environment. Odds ratios (ORs) were estimated from separate logistic regression models including one ICCE dimension at a time. All models were adjusted for baseline CDS-based atypicality, child sex, and age at baseline. ORs < 1 indicate that more favorable home-rearing environments were associated with lower risks of subsequent CDS-based atypicality.

**Table 3:** Interaction effects between ICCE dimensions and year on subsequent CDS-based atypicality.

Outcome	ICCE	Interaction OR (95% CI)	*p*-Value	FDR-Adjusted *p*
Gross motor				
	Avoidance restriction	1.000 (0.974–1.027)	0.989	0.989
	Human stimulation	0.977 (0.957–0.997)	0.028	0.044
	Social stimulation	0.965 (0.947–0.982)	<0.001	<0.001
	Social support	1.010 (0.987–1.033)	0.415	0.566
	Total ICCE score	0.984 (0.974–0.994)	0.003	0.006
Fine motor				
	Avoidance restriction	0.995 (0.974–1.017)	0.659	0.791
	Human stimulation	0.981 (0.965–0.997)	0.021	0.035
	Social stimulation	0.972 (0.957–0.987)	<0.001	0.001
	Social support	1.000 (0.981–1.019)	0.975	0.989
	Total ICCE score	0.990 (0.982–0.998)	0.012	0.022
Social competence				
	Avoidance restriction	1.007 (0.983–1.031)	0.583	0.751
	Human stimulation	0.976 (0.959–0.994)	0.008	0.015
	Social stimulation	0.967 (0.952–0.983)	<0.001	<0.001
	Social support	0.995 (0.974–1.015)	0.601	0.751
	Total ICCE score	0.988 (0.980–0.997)	0.008	0.015
Communication				
	Avoidance restriction	1.000 (0.981–1.018)	0.963	0.989
	Human stimulation	0.977 (0.964–0.991)	0.001	0.004
	Social stimulation	0.972 (0.960–0.985)	<0.001	<0.001
	Social support	0.992 (0.976–1.007)	0.290	0.415
	Total ICCE score	0.987 (0.980–0.993)	<0.001	<0.001
Vocabulary				
	Avoidance restriction	1.002 (0.986–1.017)	0.832	0.941
	Human stimulation	0.976 (0.965–0.988)	<0.001	<0.001
	Social stimulation	0.977 (0.967–0.988)	<0.001	<0.001
	Social support	0.986 (0.973–0.999)	0.031	0.047
	Total ICCE score	0.987 (0.981–0.992)	<0.001	<0.001
Intelligence				
	Avoidance restriction	0.998 (0.980–1.017)	0.847	0.941
	Human stimulation	0.970 (0.956–0.983)	<0.001	<0.001
	Social stimulation	0.970 (0.957–0.983)	<0.001	<0.001
	Social support	0.981 (0.966–0.996)	0.015	0.026
	Total ICCE score	0.983 (0.977–0.990)	<0.001	<0.001

Note: ICCE, Index of Child Care Environment. Interaction odds ratios (ORs) represent the multiplicative interaction between ICCE dimensions and calendar year in the longitudinal logistic regression models predicting CDS-based atypicality in year t + 1. Each model included one ICCE dimension, calendar year, an ICCE × year interaction term, baseline CDS-based atypicality, sex, and age.

**Table 4:** Nonlinear temporal trajectories of rolling 3-year ORs estimated using generalized additive models.

Outcome	ICCE	edf	F Value	*p*-Value
Gross motor	Avoidance Restriction	1.89	1.27	0.323
	Human stimulation	3.24	6.77	0.002
	Social stimulation	1.00	21.04	<0.001
	Social support	3.04	3.27	0.051
	Total ICCE score	1.00	13.38	0.001
Fine motor	Avoidance Restriction	2.35	2.04	0.142
	Human stimulation	2.88	4.05	0.021
	Social stimulation	2.70	5.55	0.005
	Social support	3.77	9.28	<0.001
	Total ICCE score	1.00	5.41	0.030
Social competence	Avoidance Restriction	1.00	0.82	0.375
	Human stimulation	2.00	1.54	0.313
	Social stimulation	2.99	19.50	<0.001
	Social support	2.18	2.08	0.155
	Total ICCE score	2.71	6.77	0.003
Communication	Avoidance Restriction	2.89	5.66	0.005
	Human stimulation	3.25	8.74	<0.001
	Social stimulation	3.66	10.01	<0.001
	Social support	3.44	6.09	0.003
	Total ICCE score	3.61	13.76	<0.001
Vocabulary	Avoidance Restriction	2.18	1.97	0.156
	Human stimulation	1.00	27.09	<0.001
	Social stimulation	2.18	8.35	0.001
	Social support	2.35	3.47	0.047
	Total ICCE score	2.17	15.28	<0.001
Intelligence	Avoidance Restriction	2.43	2.43	0.117
	Human stimulation	1.00	25.06	<0.001
	Social stimulation	1.64	10.87	<0.001
	Social support	1.00	3.65	0.069
	Total ICCE score	3.52	14.36	<0.001

Note: ICCE, Index of Child Care Environment. Generalized additive models (GAMs) were fitted to rolling 3-year odds ratios (ORs) estimated from longitudinal logistic regression analyses examining associations between ICCE dimensions and subsequent CDS-based atypicality. Estimated degrees of freedom (edf) > 1 indicate nonlinear temporal trajectories of OR changes across the study period.

## Data Availability

No copyrighted material was used in this study. Data supporting the findings of this study are available upon request from the corresponding author. The data are not publicly available because of privacy or ethical restrictions.

## Supplementary Materials

The following supporting information can be downloaded at https://www.mdpi.com/article/10.3390/bs16081427/s1, Table S1. Ordinal Alpha Coefficients for ICCE Domains, Overall and by Two Periods. Table S2. Multi-Group CFA Model Fit and Invariance Comparison. Table S3. Annual demographic characteristics of the study population. Table S4. Annual mean (SD) baseline and follow-up the Index of Child Care Environment scores of the study population. Table S5. Annual baseline and 1-year follow-up prevalence of child developmental atypicality. Table S6. Annual valid sample sizes for the Index of Child Care Environment and Child development scale variables. Table S7. Annual valid sample sizes for the Child development scale variables. Table S8. Cluster-Robust Sensitivity Analysis of the Main Associations. Table S9. Cluster-Robust Sensitivity Analysis of the Calendar-Year Interaction Associations. File S2. Rolling 3-year and annual logistic regression estimates for the associations between caregiving environments and developmental atypicality.

## Abbreviations

The following abbreviations are used in this manuscript:

ICCE	Index of Child Care Environment
CDS	Child Development Scale
OR	Odds ratio
CI	Confidence interval
GAM	Generalized additive model

## References

[B1-behavsci-16-01427] Alonso J., Little E. (2019). Parent help-seeking behaviour: Examining the impact of parent beliefs on professional help-seeking for child emotional and behavioural difficulties. The Educational and Developmental Psychologist.

[B2-behavsci-16-01427] Andersson M. A. (2016). Health returns to education by family socioeconomic origins, 1980–2008: Testing the importance of gender, cohort, and age. SSM–Population Health.

[B3-behavsci-16-01427] Anme T., Segal U. A. (2004). Implications for the development of children in over 11 h of centre-based care. Child: Care, Health and Development.

[B4-behavsci-16-01427] Anme T., Segal U. A. (2007). Implications of japan’s center-based night care: A 1-year follow-up. Early Childhood Education Journal.

[B5-behavsci-16-01427] Anme T., Shinohara R., Sugisawa Y. (2007). Development of a developmental checklist for childcare children based on a nationwide survey of child development in Japan.

[B6-behavsci-16-01427] Anme T., Tanaka E., Watanabe T., Tomisaki E., Mochizuki Y., Tokutake K. (2013). Validity and reliability of the index of child care environment (ICCE). Journal of Public Health Frontier.

[B7-behavsci-16-01427] Ben Amor L., Imene L., Mohamed J. (2016). Recursive and rolling windows for medical time series forecasting: A comparative study. 2016 IEEE Intl Conference on Computational Science and Engineering (CSE) and IEEE Intl Conference on Embedded and Ubiquitous Computing (EUC) and 15th Intl Symposium on Distributed Computing and Applications for Business Engineering (DCABES).

[B8-behavsci-16-01427] Benjamini Y., Hochberg Y. (1995). Controlling the false discovery rate: A practical and powerful approach to multiple testing. Journal of the Royal Statistical Society Series B: Statistical Methodology.

[B9-behavsci-16-01427] Braune-Krickau K., Schneebeli L., Pehlke-Milde J., Gemperle M., Koch R., von Wyl A. (2021). Smartphones in the nursery: Parental smartphone use and parental sensitivity and responsiveness within parent–child interaction in early childhood (0–5 years): A scoping review. Infant Mental Health Journal: Infancy and Early Childhood.

[B10-behavsci-16-01427] Britto P. R., Lye S. J., Proulx K., Yousafzai A. K., Matthews S. G., Vaivada T., Perez-Escamilla R., Rao N., Ip P., Fernald L. C. H., MacMillan H., Hanson M., Wachs T. D., Yao H., Yoshikawa H., Cerezo A., Leckman J. F., Bhutta Z. A. (2017). Nurturing care: Promoting early childhood development. The Lancet.

[B11-behavsci-16-01427] Bronfenbrenner U. (2000). Ecological systems theory.

[B12-behavsci-16-01427] Brown A., Council on Communications and Media (2011). Media use by children younger than 2 years. Pediatrics.

[B13-behavsci-16-01427] Brushe M. E., Haag D. G., Melhuish E. C., Reilly S., Gregory T. (2024). Screen time and parent-child talk when children are aged 12 to 36 months. JAMA Pediatrics.

[B14-behavsci-16-01427] Buck D. M., Richmond V., Brown K. H., Chödrön G. S., Center W. (2022). Promoting family engagement and early identification of developmental delays: The role of act early ambassadors.

[B15-behavsci-16-01427] Cadarso-Suárez C., Roca-Pardiñas J., Figueiras A., González-Manteiga W. (2005). Non-parametric estimation of the odds ratios for continuous exposures using generalized additive models with an unknown link function. Statistics in Medicine.

[B16-behavsci-16-01427] Ottawa O., Canadian Paediatric Society, Digital Health Task Force (2017). Screen time and young children: Promoting health and development in a digital world. Paediatrics & Child Health.

[B17-behavsci-16-01427] Chen F. F. (2007). Sensitivity of goodness of fit indexes to lack of measurement invariance. Structural Equation Modeling: A Multidisciplinary Journal.

[B18-behavsci-16-01427] Children and Families Agency (2019). Free early childhood education and care.

[B19-behavsci-16-01427] Christensen R., Miller S. P., Gomaa N. A. (2024). Home-ics: How experiences of the home impact biology and child neurodevelopmental outcomes. Pediatric Research.

[B20-behavsci-16-01427] Coffey J. R., Snedeker J. (2026). How strong is the relationship between caregiver speech and language development? A meta-analysis. Journal of Child Language.

[B21-behavsci-16-01427] Collishaw S., Gardner F., Maughan B., Scott J., Pickles A. (2012). Do historical changes in parent–child relationships explain increases in youth conduct problems?. Journal of Abnormal Child Psychology.

[B22-behavsci-16-01427] Conti G., Heckman J. J. (2010). Understanding the early origins of the education–health gradient: A framework that can also be applied to analyze gene–environment interactions. Perspectives on Psychological Science.

[B23-behavsci-16-01427] Dawson G., Rogers S., Munson J., Smith M., Winter J., Greenson J., Donaldson A., Varley J. (2010). Randomized, controlled trial of an intervention for toddlers with autism: The early start denver model. Pediatrics.

[B24-behavsci-16-01427] Dentsu (2015). Japanese people and smartphones.

[B25-behavsci-16-01427] Fish L., Bernardi M., van de Grint-Stoop J., Heng J., Knibbs S., Goodman A., Calderwood L., Mathers S., Deepchand K., Ferguson C. (2026). Children of the 2020s: Home learning environment and screen time at age 2.

[B26-behavsci-16-01427] Gadermann A., Guhn M., Zumbo B. D. (2014). Ordinal alpha.

[B27-behavsci-16-01427] Ghani N., Jamian A. R., Jobar N. A. (2022). The relationship between language acquisition and theory of social interactionist. ICCCM Journal of Social Sciences and Humanities.

[B28-behavsci-16-01427] Glickman M. E., Rao S. R., Schultz M. R. (2014). False discovery rate control is a recommended alternative to bonferroni-type adjustments in health studies. Journal of Clinical Epidemiology.

[B29-behavsci-16-01427] Government of Japan (2012). Children and child-rearing support act.

[B30-behavsci-16-01427] Halfon N., Larson K., Lu M., Tullis E., Russ S. (2014). Lifecourse health development: Past, present and future. Maternal and Child Health Journal.

[B31-behavsci-16-01427] Hamad R., Rehkopf D. H. (2016). Poverty and child development: A longitudinal study of the impact of the earned income tax credit. American Journal of Epidemiology.

[B32-behavsci-16-01427] Harverson J., Paatsch L., Anglim J., Horwood S. (2025). Digital technology use and well-being in young children: A systematic review and meta-analysis. Computers in Human Behavior.

[B33-behavsci-16-01427] Huang F. L., Zhang B., Li X. (2023). Using robust standard errors for the analysis of binary outcomes with a small number of clusters. Journal of Research on Educational Effectiveness.

[B34-behavsci-16-01427] Jeong J., Franchett E. E., Ramos de Oliveira C. V., Rehmani K., Yousafzai A. K. (2021). Parenting interventions to promote early child development in the first three years of life: A global systematic review and meta-analysis. PLoS Medicine.

[B35-behavsci-16-01427] Kreski N. T., Riehm K. E., Cerdá M., Chen Q., Hasin D. S., Martins S. S., Mauro P. M., Olfson M., Keyes K. M. (2023). Parenting practices and adolescent internalizing symptoms in the united states, 1991–2019. Journal of Adolescent Health.

[B36-behavsci-16-01427] Lam C. M., Kwong W.-M., To S.-M. (2019). Has parenting changed over past decade? A qualitative study of generational shifts in parenting. International Journal of Social Science and Humanity.

[B37-behavsci-16-01427] Lebrun-Harris L. A., Ghandour R. M., Kogan M. D., Warren M. D. (2022). Five-Year trends in US children’s health and well-being, 2016-2020. JAMA Pediatrics.

[B38-behavsci-16-01427] Leeb R. T., Danielson M. L., Claussen A. H., Robinson L. R., Lebrun-Harris L. A., Ghandour R., Bitsko R. H., Katz S. M., Kaminski J. W., Brown J. (2024). Trends in mental, behavioral, and developmental disorders among children and adolescents in the US, 2016–2021. Preventing Chronic Disease.

[B39-behavsci-16-01427] Li X., Jiao D., Matsumoto M., Zhu Y., Zhang J., Zhu Z., Liu Y., Cui M., Wang Y., Qian M. (2022). Home environment and social skills of Japanese preschool children pre-and post-COVID-19. Early Child Development and Care.

[B40-behavsci-16-01427] Lipkin P. H., Macias M. M., Baer Chen B., Coury D., Gottschlich E. A., Hyman S. L., Sisk B., Wolfe A., Levy S. E. (2020). Trends in pediatricians’ developmental screening: 2002–2016. Pediatrics.

[B41-behavsci-16-01427] Luef E. M. (2018). The talking species: Perspectives on the evolutionary, neuronal and cultural foundations of language.

[B42-behavsci-16-01427] Lurie L. A., Hagen M. P., McLaughlin K. A., Sheridan M. A., Meltzoff A. N., Rosen M. L. (2021). Mechanisms linking socioeconomic status and academic achievement in early childhood: Cognitive stimulation and language. Cognitive Development.

[B43-behavsci-16-01427] Mallawaarachchi S., Burley J., Mavilidi M., Howard S. J., Straker L., Kervin L., Staton S., Hayes N., Machell A., Torjinski M., Brady B., Thomas G., Horwood S., White S. L. J., Zabatiero J., Rivera C., Cliff D. (2024). Early childhood screen use contexts and cognitive and psychosocial outcomes. JAMA Pediatrics.

[B44-behavsci-16-01427] Mann S., Calvin A., Lenhart A., Robb M. (2025). The common sense census: Media use by kids zero to eight.

[B45-behavsci-16-01427] Marek S., Donohue M. R., Karcher N. R., Hoyniak C. P., Chauvin R. J., Meyer A. C., Miller J., Van A. N., Wang A., Baden N. J., Suljic V., Scheidter K. M., Monk J., Whiting F. I., Ramirez-Perez N. J., Krimmel S. R., Metoki A., Paul S. E., Gorelik A. J., Dosenbach N. U. F. (2026). Patterns of brain-wide associations reflect socioeconomics. Science.

[B46-behavsci-16-01427] McArthur B. A., Browne D., McDonald S., Tough S., Madigan S. (2021). Longitudinal associations between screen use and reading in preschool-aged children. Pediatrics.

[B47-behavsci-16-01427] Merçon-Vargas E. A., Lima R. F. F., Rosa E. M., Tudge J. (2020). Processing proximal processes: What bronfenbrenner meant, what he didn’t mean, and what he should have meant. Journal of Family Theory & Review.

[B48-behavsci-16-01427] Munzer T., Parga-Belinkie J., Milkovich L. M., Tomopoulos S., Ajumobi T., Cross C., Gerwin R., Madigan S., Psych R., Radesky J., Evans Y., Cahan E., Milkovich E., Bracho-Sanchez E., Parga-Belinkie J., Munzer T., Espinoza J., Husain A., Patel A., Shapiro I. (2026). Digital ecosystems, children, and adolescents: Policy statement. Pediatrics.

[B49-behavsci-16-01427] OECD (2025). How’s life for children in the digital age?.

[B50-behavsci-16-01427] OECD (2026). Building strong foundations for life: Results from the 2025 early learning and child well-being study.

[B51-behavsci-16-01427] O’Keefe P., Rodgers J. L. (2022). Home improvement: Evaluating secular changes in NLSY HOME-cognitive stimulation and emotional support scores. Journal of Child and Family Studies.

[B52-behavsci-16-01427] Olusanya B. O., Smythe T., Ogbo F. A., Nair M. K. C., Scher M., Davis A. C. (2023). Global prevalence of developmental disabilities in children and adolescents: A systematic umbrella review. Front Public Health.

[B53-behavsci-16-01427] Oshio T. (2020). Lingering impact of starting working life during a recession: Health outcomes of survivors of the “employment ice age” (1993–2004) in Japan. Journal of Epidemiology.

[B54-behavsci-16-01427] Racine N., Eirich R., Cooke J., Zhu J., Pador P., Dunnewold N., Madigan S. (2022). When the bough breaks: A systematic review and meta-analysis of mental health symptoms in mothers of young children during the COVID-19 pandemic. Infant Mental Health Journal.

[B55-behavsci-16-01427] Sawrikar V., Van Dyke C., Smith Slep A. M. (2026). The ws of parental help-seeking: When, where, and for what do parents seek help for child mental health. Child Psychiatry and Human Development.

[B56-behavsci-16-01427] Schlesinger Y., Paz Y., Rousseau S., Atzaba-Poria N., Frenkel T. I. (2025). The emotional vaccine: Maternal caregiving in infancy shaped future preschoolers’ internalizing symptoms during the COVID-19 pandemic. Child Development.

[B57-behavsci-16-01427] Schoellman T. (2016). Early childhood human capital and development. American Economic Journal: Macroeconomics.

[B58-behavsci-16-01427] Settels J. (2022). Further resource multiplication at more advanced ages? Interactions between education, parental socioeconomic status, and age in their impacts upon health. Sociology Compass.

[B59-behavsci-16-01427] Sigerson L., Li A. Y. L., Cheung M. W. L., Luk J. W., Cheng C. (2017). Psychometric properties of the Chinese internet gaming disorder scale. Addictive Behaviors.

[B60-behavsci-16-01427] Sugiyama M., Tsuchiya K. J., Okubo Y., Rahman M. S., Uchiyama S., Harada T., Iwabuchi T., Okumura A., Nakayasu C., Amma Y., Suzuki H., Takahashi N., Kinsella-Kammerer B., Nomura Y., Itoh H., Nishimura T. (2023). Outdoor play as a mitigating factor in the association between screen time for young children and neurodevelopmental outcomes. JAMA Pediatrics.

[B61-behavsci-16-01427] Sum K. K., Cai S., Law E., Cheon B., Tan G., Loo E., Lee Y. S., Yap F., Chan J. K. Y., Daniel M., Chong Y. S., Meaney M., Eriksson J., Huang J. (2022). COVID-19–Related life experiences, outdoor play, and long-term adiposity changes among preschool- and school-aged children in Singapore 1 year after lockdown. JAMA Pediatrics.

[B62-behavsci-16-01427] Svetina D., Rutkowski L., Rutkowski D. (2020). Multiple-group invariance with categorical outcomes using updated guidelines: An illustration using M plus and the lavaan/semtools packages. Structural Equation Modeling: A Multidisciplinary Journal.

[B63-behavsci-16-01427] Takahashi I., Obara T., Ishikuro M., Murakami K., Ueno F., Noda A., Onuma T., Shinoda G., Nishimura T., Tsuchiya K. J. (2023). Screen time at age 1 year and communication and problem-solving developmental delay at 2 and 4 years. JAMA Pediatrics.

[B64-behavsci-16-01427] Takahashi Y., Okada K., Hoshino T., Anme T. (2008). Social skills of preschoolers: Stability of factor structures and predictive validity from a nationwide cohort study in Japan. Japanese Journal of Educational Psychology.

[B65-behavsci-16-01427] The Japan Ministry of Health, Labour and Welfare (2004). Act on support for persons with developmental disabilities (Act No. 167 of 2004).

[B66-behavsci-16-01427] Toledo-Vargas M., Chong K. H., Maddren C. I., Howard S. J., Wakefield B., Okely A. D. (2025). Parental technology use in a child’s presence and health and development in the early years: A systematic review and meta-analysis. JAMA Pediatrics.

[B67-behavsci-16-01427] Victora C. G., Hartwig F. P., Vidaletti L. P., Martorell R., Osmond C., Richter L. M., Stein A. D., Barros A. J. D., Adair L. S., Barros F. C., Bhargava S. K., Horta B. L., Kroker-Lobos M. F., Lee N. R., Menezes A. M. B., Murray J., Norris S. A., Sachdev H. S., Stein A., Black R. E. (2022). Effects of early-life poverty on health and human capital in children and adolescents: Analyses of national surveys and birth cohort studies in LMICs. Lancet.

[B68-behavsci-16-01427] Wickham S., Whitehead M., Taylor-Robinson D., Barr B. (2017). The effect of a transition into poverty on child and maternal mental health: A longitudinal analysis of the UK millennium cohort study. The Lancet Public Health.

[B69-behavsci-16-01427] Wood S. N. (2011). Fast stable restricted maximum likelihood and marginal likelihood estimation of semiparametric generalized linear models. Journal of the Royal Statistical Society Series B: Statistical Methodology.

[B70-behavsci-16-01427] World Health Organization (2020). Improving early childhood development: WHO guideline.

[B71-behavsci-16-01427] Wu X., Cheng G., Tang C., Xie Q., He S., Ruotong L., Yan Y. (2020). The effect of parenting quality on child development at 36–48 months in china’s urban area: Evidence from a birth cohort study. International Journal of Environmental Research and Public Health.

[B72-behavsci-16-01427] Yang Q., Yang J., Zheng L., Song W., Yi L. (2021). Impact of home parenting environment on cognitive and psychomotor development in children under 5 years old: A meta-analysis. Frontiers in Pediatrics.

[B73-behavsci-16-01427] Zeng N., Ayyub M., Sun H., Wen X., Xiang P., Gao Z. (2017). Effects of physical activity on motor skills and cognitive development in early childhood: A systematic review. BioMed Research International.

[B74-behavsci-16-01427] Zhu Z., Tanaka E., Tomisaki E., Watanabe T., Sawada Y., Li X., Jiao D., Ajmal A., Matsumoto M., Zhu Y., Anme T. (2021). Do it yourself: The role of early self-care ability in social skills in Japanese preschool settings. School Psychology International.

[B75-behavsci-16-01427] Zivot E., Wang J. (2003). Rolling analysis of time series. Modeling financial time series with S-plus^®^.

[B76-behavsci-16-01427] Zumbo B. D., Gadermann A. M., Zeisser C. (2007). Ordinal versions of coefficients alpha and theta for likert rating scales. Journal of Modern Applied Statistical Methods.

