# Supplementary material for: Changing Longitudinal Associations Between Home-Rearing Environments and Child Developmental Atypicality Across Two Decades
Source: Behav Sci (Basel). 2026 Aug 19;16(8):1427. doi: 10.3390/bs16081427 (PMC13509869; doi:10.3390/bs16081427)
Supplement: Supplementary file 1 [file behavsci-16-01427-s001.zip › 0814 behavsci-4445811 File S1.pdf]

Table S1. Ordinal Alpha Coefficients for ICCE Domains by Period

Domain	No. items	Overall Ordinal α	Before 2010 Ordinal α	2010 or later Ordinal α
Avoidance restriction	2	0.691	0.641	0.706
Human stimulation	5	0.744	0.767	0.716
Social stimulation	3	0.546	0.540	0.559
Social support	3	0.663	0.665	0.655
Total ICCE	13	0.800	0.810	0.770

Table S2. Multi-Group CFA Model Fit and Invariance Comparison

Model	χ^2	df	CFI	TLI	RMSEA	SRMR	ΔCFI	ΔRMSEA	ΔSRMR
Configural	2848.44	118	0.863	0.819	0.047	0.112	—	—	—
Loading-constrained	2725.73	127	0.869	0.84	0.044	0.116	0.007	−0.003	0.004

Table S3. Annual demographic characteristics of the study population.

Category	Sample size	Sex, n (%)		Age group, n (%)	
		Girls	Boys	0–3 years	4–6 years
**1999**	2317	1144 (49.4)	1173 (50.6)	1297 (60.4)	852 (39.6)
**2000**	2411	1165 (48.3)	1246 (51.7)	1364 (59.6)	925 (40.4)
**2001**	2303	1137 (49.4)	1166 (50.6)	1306 (59.1)	904 (40.9)
**2002**	2311	1116 (49.0)	1161 (51.0)	1301 (58.4)	925 (41.6)
**2003**	2191	1064 (48.6)	1127 (51.4)	1271 (59.2)	876 (40.8)
**2004**	1579	751 (47.6)	828 (52.4)	929 (59.4)	636 (40.6)
**2005**	1361	652 (51.0)	627 (49.0)	799 (58.7)	562 (41.3)
**2006**	1511	746 (49.4)	763 (50.6)	881 (59.2)	607 (40.8)
**2007**	1353	628 (46.9)	711 (53.1)	819 (61.3)	518 (38.7)
**2008**	1221	567 (47.0)	639 (53.0)	746 (61.8)	461 (38.2)
**2009**	1027	478 (47.7)	525 (52.3)	644 (63.4)	371 (36.6)
**2010**	435	204 (47.7)	224 (52.3)	268 (62.0)	164 (38.0)
**2011**	632	315 (49.8)	317 (50.2)	382 (60.7)	247 (39.3)
**2012**	1356	682 (50.4)	671 (49.6)	711 (53.7)	614 (46.3)
**2013**	1670	817 (49.0)	851 (51.0)	756 (47.4)	838 (52.6)
**2014**	1709	832 (48.7)	875 (51.3)	893 (57.0)	673 (43.0)
**2015**	1690	786 (46.5)	904 (53.5)	895 (54.9)	734 (45.1)
**2016**	1802	838 (46.5)	964 (53.5)	913 (55.5)	731 (44.5)
**2017**	1977	919 (46.5)	1056 (53.5)	1020 (54.0)	868 (46.0)
**2018**	1680	775 (46.1)	905 (53.9)	894 (55.4)	721 (44.6)
**2019**	1467	657 (44.8)	810 (55.2)	758 (52.5)	686 (47.5)
**2020**	1699	773 (45.5)	926 (54.5)	878 (53.3)	770 (46.7)
**2021**	1676	799 (47.7)	877 (52.3)	854 (53.2)	752 (46.8)
**2022**	1593	733 (46.0)	860 (54.0)	860 (56.0)	677 (44.0)
**2023**	1656	762 (46.0)	894 (54.0)	781 (48.6)	826 (51.4)

Table S4. Annual mean (SD) baseline and follow-up the Index of Child Care Environment scores of the study population

Year	Baseline ICCE score, mean (SD)					Follow-up ICCE score, mean (SD)				
	AR	HS	SST	SSU	Total ICCE	AR	HS	SST	SSU	Total ICCE
**1999**	1.49 (0.57)	4.71 (0.65)	2.25 (0.76)	2.60 (0.64)	11.03 (1.58)	1.44 (0.58)	4.66 (0.68)	2.32 (0.72)	2.62 (0.66)	11.06 (1.56)
**2000**	1.47 (0.57)	4.68 (0.64)	2.31 (0.72)	2.64 (0.63)	11.09 (1.53)	1.48 (0.59)	4.70 (0.66)	2.30 (0.73)	2.64 (0.60)	11.13 (1.58)
**2001**	1.46 (0.60)	4.69 (0.67)	2.26 (0.76)	2.64 (0.60)	11.08 (1.57)	1.47 (0.58)	4.66 (0.70)	2.24 (0.77)	2.63 (0.63)	11.01 (1.64)
**2002**	1.49 (0.58)	4.67 (0.74)	2.23 (0.77)	2.64 (0.63)	11.03 (1.70)	1.48 (0.60)	4.70 (0.67)	2.32 (0.74)	2.69 (0.57)	11.24 (1.59)
**2003**	1.50 (0.60)	4.71 (0.63)	2.26 (0.76)	2.69 (0.57)	11.19 (1.53)	1.48 (0.61)	4.70 (0.65)	2.36 (0.72)	2.66 (0.61)	11.21 (1.59)
**2004**	1.48 (0.62)	4.72 (0.62)	2.36 (0.72)	2.65 (0.62)	11.24 (1.60)	1.53 (0.59)	4.68 (0.65)	2.27 (0.73)	2.66 (0.63)	11.18 (1.50)
**2005**	1.55 (0.58)	4.71 (0.59)	2.24 (0.75)	2.66 (0.60)	11.20 (1.44)	1.57 (0.59)	4.68 (0.62)	2.34 (0.70)	2.68 (0.60)	11.29 (1.46)
**2006**	1.56 (0.58)	4.74 (0.55)	2.35 (0.69)	2.70 (0.58)	11.36 (1.41)	1.56 (0.57)	4.73 (0.58)	2.39 (0.70)	2.71 (0.59)	11.42 (1.46)
**2007**	1.55 (0.57)	4.76 (0.56)	2.33 (0.72)	2.72 (0.59)	11.40 (1.43)	1.47 (0.60)	4.70 (0.62)	2.40 (0.69)	2.74 (0.55)	11.30 (1.48)
**2008**	1.50 (0.58)	4.72 (0.59)	2.41 (0.70)	2.73 (0.56)	11.37 (1.41)	1.47 (0.56)	4.73 (0.59)	2.46 (0.67)	2.74 (0.53)	11.39 (1.35)
**2009**	1.51 (0.55)	4.77 (0.56)	2.45 (0.67)	2.71 (0.54)	11.47 (1.31)	1.45 (0.60)	4.75 (0.57)	2.51 (0.65)	2.79 (0.47)	11.57 (1.25)
**2010**	1.51 (0.52)	4.66 (0.76)	2.46 (0.67)	2.69 (0.54)	11.35 (1.68)	1.54 (0.62)	4.68 (0.68)	2.33 (0.80)	2.66 (0.63)	11.18 (1.79)
**2011**	1.51 (0.61)	4.66 (0.67)	2.33 (0.72)	2.63 (0.67)	11.08 (1.51)	1.61 (0.56)	4.64 (0.68)	2.42 (0.70)	2.74 (0.53)	11.42 (1.51)
**2012**	1.62 (0.55)	4.74 (0.55)	2.42 (0.71)	2.73 (0.54)	11.53 (1.37)	1.62 (0.55)	4.70 (0.60)	2.41 (0.70)	2.72 (0.56)	11.46 (1.32)
**2013**	1.65 (0.53)	4.71 (0.61)	2.39 (0.72)	2.72 (0.56)	11.49 (1.37)	1.66 (0.52)	4.68 (0.60)	2.45 (0.70)	2.72 (0.55)	11.54 (1.34)
**2014**	1.67 (0.52)	4.72 (0.56)	2.46 (0.69)	2.72 (0.54)	11.57 (1.34)	1.64 (0.54)	4.68 (0.63)	2.53 (0.63)	2.73 (0.55)	11.57 (1.35)
**2015**	1.67 (0.51)	4.72 (0.60)	2.50 (0.64)	2.73 (0.54)	11.62 (1.31)	1.68 (0.49)	4.73 (0.59)	2.54 (0.63)	2.77 (0.52)	11.76 (1.31)
**2016**	1.70 (0.48)	4.75 (0.57)	2.52 (0.63)	2.76 (0.52)	11.76 (1.27)	1.68 (0.50)	4.68 (0.65)	2.49 (0.66)	2.69 (0.58)	11.56 (1.44)
**2017**	1.72 (0.49)	4.73 (0.60)	2.49 (0.65)	2.70 (0.58)	11.65 (1.40)	1.74 (0.47)	4.64 (0.68)	2.50 (0.63)	2.71 (0.57)	11.61 (1.45)
**2018**	1.73 (0.47)	4.67 (0.65)	2.49 (0.64)	2.72 (0.57)	11.64 (1.38)	1.73 (0.48)	4.70 (0.59)	2.51 (0.62)	2.76 (0.53)	11.71 (1.32)
**2019**	1.77 (0.44)	4.75 (0.57)	2.50 (0.65)	2.77 (0.51)	11.79 (1.30)	1.80 (0.42)	4.69 (0.60)	2.42 (0.67)	2.72 (0.54)	11.64 (1.31)
**2020**	1.86 (0.37)	4.73 (0.59)	2.40 (0.68)	2.73 (0.55)	11.72 (1.31)	1.84 (0.39)	4.70 (0.60)	2.40 (0.65)	2.71 (0.56)	11.65 (1.32)
**2021**	1.86 (0.36)	4.74 (0.56)	2.37 (0.66)	2.68 (0.57)	11.65 (1.26)	1.86 (0.35)	4.66 (0.65)	2.42 (0.64)	2.72 (0.55)	11.65 (1.33)
**2022**	1.87 (0.35)	4.71 (0.60)	2.41 (0.65)	2.73 (0.54)	11.71 (1.31)	1.88 (0.34)	4.69 (0.62)	2.46 (0.63)	2.77 (0.53)	11.82 (1.27)
**2023**	1.90 (0.32)	4.75 (0.54)	2.41 (0.62)	2.73 (0.55)	11.81 (1.20)	1.88 (0.34)	4.67 (0.64)	2.44 (0.61)	2.79 (0.50)	11.79 (1.29)

Note: AR = Avoidance restriction; HS = Human Stimulation; SST = Social Stimulation; SSU = Social Support; ICCE = Index of Child Care Environment.

Table S5. Annual baseline and 1-year follow-up prevalence of child developmental atypicality

Year	Baseline CDS-based atypicality, (%, 95% CI)												Follow-up CDS-based atypicality, (%, 95% CI)											
	Gross motor		Fine motor		Social competence		Communication		Vocabulary		Intelligence		Gross motor		Fine motor		Social competence		Communication		Vocabulary		Intelligence	
	%	CI	%	CI	%	CI	%	CI	%	CI	%	CI	%	CI	%	CI	%	CI	%	CI	%	CI	%	CI
**1999**	8.2	7.0 – 9.4	9.4	8.2 – 10.7	7.7	6.6 – 9.0	8.6	7.5 – 9.9	11.6	10.3 – 13.1	8.7	7.6 – 10.0	2.7	2.1 – 3.5	1.0	0.7 – 1.6	0.7	0.4 – 1.1	1.7	1.2 – 2.4	2.5	1.9 – 3.2	1.3	0.9 – 1.9
**2000**	3.8	3.1 – 4.7	1.3	0.9 – 1.9	1.3	0.9 – 1.9	1.9	1.4 – 2.6	3.7	3.0 – 4.6	1.8	1.3 – 2.5	2.0	1.5 – 2.7	1.7	1.2 – 2.4	1.3	0.9 – 1.9	2.2	1.6 – 2.9	3.2	2.5 – 4.0	1.9	1.4 – 2.6
**2001**	2.2	1.7 – 3.0	1.6	1.1 – 2.2	1.4	1.0 – 2.0	2.0	1.4 – 2.7	5.4	4.5 – 6.4	1.7	1.3 – 2.4	1.2	0.8 – 1.8	1.2	0.8 – 1.8	0.5	0.2 – 0.9	1.7	1.2 – 2.4	2.8	2.1 – 3.6	0.8	0.5 – 1.3
**2002**	1.3	0.9 – 1.9	1.4	0.9 – 2.0	1.0	0.6 – 1.5	2.2	1.7 – 2.9	4.0	3.3 – 5.0	1.2	0.8 – 1.8	1.3	0.9 – 1.8	0.8	0.5 – 1.3	0.4	0.2 – 0.9	1.5	1.0 – 2.1	3.3	2.6 – 4.1	1.3	0.9 – 1.8
**2003**	1.3	0.9 – 1.9	1.1	0.7 – 1.6	0.7	0.4 – 1.1	2.1	1.6 – 2.8	4.8	4.0 – 5.8	1.2	0.8 – 1.8	1.2	0.8 – 1.8	0.9	0.6 – 1.5	0.5	0.3 – 0.9	1.6	1.1 – 2.2	2.7	2.1 – 3.5	1.4	1.0 – 2.0
**2004**	0.9	0.5 – 1.5	1.3	0.8 – 2.0	1.0	0.6 – 1.7	2.2	1.5 – 3.1	5.2	4.2 – 6.4	1.8	1.2 – 2.6	0.2	0 – 0.6	27.2	25.0 – 29.5	32.2	29.9 – 34.6	31.6	29.3 – 34.0	40.5	38.0 – 43.0	42.8	40.3 – 45.3
**2005**	0.4	0.1 – 0.9	16.5	14.6 – 18.6	24.3	22.0 – 26.7	21.6	19.4 – 23.9	29.6	27.2 – 32.1	30.8	28.3 – 33.3	0.4	0.1 – 0.9	0.3	0.1 – 0.8	0.1	0 – 0.6	0.9	0.5 – 1.6	1.6	1.1 – 2.5	0.7	0.3 – 1.3
**2006**	0.3	0.1 – 0.8	0.5	0.2 – 1.0	0.3	0.1 – 0.8	1.5	1.0 – 2.3	2.6	1.9 – 3.6	0.7	0.4 – 1.4	0.6	0.3 – 1.2	0.4	0.2 – 0.9	0.4	0.2 – 0.9	1.1	0.7 – 1.9	1.7	1.2 – 2.6	0.7	0.3 – 1.3
**2007**	1.0	0.6 – 1.8	1.1	0.6 – 1.9	1.3	0.8 – 2.1	2.3	1.6 – 3.3	3.6	2.6 – 4.8	1.6	1.0 – 2.5	1.5	1.0 – 2.4	1.4	0.8 – 2.2	1.1	0.6 – 1.8	2.8	2.0 – 3.9	3.9	2.9 – 5.1	1.8	1.1 – 2.7
**2008**	1.2	0.7 – 2.0	2.2	1.4 – 3.2	2.3	1.6 – 3.4	3.2	2.3 – 4.5	6.4	5.1 – 8.0	2.4	1.7 – 3.5	1.2	0.7 – 2.1	0.8	0.4 – 1.6	0.6	0.3 – 1.2	2.2	1.4 – 3.2	3.1	2.2 – 4.3	1.7	1.1 – 2.7
**2009**	2.1	1.3 – 3.2	2.5	1.7 – 3.7	2.4	1.6 – 3.6	4.6	3.4 – 6.1	5.9	4.6 – 7.6	1.8	1.1 – 2.9	1.9	1.2 – 3.0	1.4	0.8 – 2.4	0.6	0.2 – 1.4	2.6	1.7 – 3.8	3.6	2.6 – 5.0	2.3	1.5 – 3.5
**2010**	2.1	1.0 – 4.1	1.9	0.9 – 3.8	1.9	0.9 – 3.8	4.9	3.1 – 7.5	5.9	3.9 – 8.6	2.8	1.5 – 5.0	3.6	2.1 – 6.1	1.2	0.4 – 3.2	1.2	0.4 – 3.2	1.2	0.4 – 3.3	3.3	1.7 – 6.0	1.5	0.6 – 3.7
**2011**	4.3	2.9 – 6.3	3.3	2.0 – 5.3	2.4	1.3 – 4.2	2.2	1.2 – 4.0	5.9	4.2 – 8.4	2.6	1.5 – 4.4	2.4	1.4 – 4.2	4.6	3.1 – 6.7	2.3	1.3 – 3.9	5.3	3.7 – 7.5	7.9	6.0 – 10.4	3.5	2.2 – 5.3
**2012**	1.9	1.3 – 2.9	1.6	1.0 – 2.5	2.1	1.5 – 3.1	3.6	2.7 – 4.8	5.3	4.2 – 6.7	2.5	1.8 – 3.6	2.9	2.1 – 4.1	3.7	2.7 – 4.9	2.8	2.0 – 3.9	4.1	3.1 – 5.3	5.1	4.0 – 6.5	3.6	2.7 – 4.9
**2013**	3.1	2.3 – 4.1	3.7	2.9 – 4.8	2.8	2.1 – 3.8	4.8	3.8 – 6.0	7.0	5.8 – 8.4	3.3	2.5 – 4.3	3.5	2.7 – 4.6	2.4	1.8 – 3.4	2.2	1.6 – 3.1	4.1	3.2 – 5.2	5.3	4.2 – 6.5	2.0	1.4 – 2.9
**2014**	4.4	3.5 – 5.6	3.2	2.4 – 4.3	3.1	2.3 – 4.1	5.3	4.3 – 6.6	7.9	6.6 – 9.4	2.9	2.1 – 3.9	2.0	1.4 – 2.8	2.4	1.7 – 3.3	1.4	0.9 – 2.2	3.8	2.9 – 4.9	4.9	3.9 – 6.1	2.8	2.1 – 3.8
**2015**	3.0	2.3 – 4.0	3.1	2.4 – 4.1	2.8	2.0 – 3.7	4.6	3.7 – 5.8	7.4	6.2 – 8.9	4.2	3.3 – 5.3	2.6	1.9 – 3.6	1.9	1.3 – 2.7	1.3	0.8 – 2.0	3.2	2.4 – 4.2	5.3	4.2 – 6.6	2.8	2.1 – 3.8
**2016**	2.6	1.9 – 3.5	2.4	1.7 – 3.3	2.9	2.1 – 3.8	4.0	3.2 – 5.1	7.4	6.2 – 8.8	2.5	1.8 – 3.4	3.5	2.7 – 4.5	3.0	2.3 – 3.9	2.0	1.4 – 2.8	4.8	3.8 – 5.9	6.5	5.4 – 7.8	3.8	3.0 – 4.9
**2017**	4.0	3.2 – 5.0	4.9	4.0 – 6.0	4.8	3.9 – 5.9	6.0	4.9 – 7.1	9.0	7.8 – 10.5	4.6	3.7 – 5.7	1.5	1.0 – 2.2	1.5	1.0 – 2.2	2.2	1.6 – 3.0	2.8	2.1 – 3.7	3.8	3.0 – 4.8	2.1	1.5 – 2.8
**2018**	1.7	1.2 – 2.5	2.0	1.4 – 2.8	2.9	2.1 – 3.8	3.5	2.7 – 4.6	5.4	4.4 – 6.7	2.7	2.0 – 3.6	2.6	1.9 – 3.6	1.9	1.3 – 2.7	1.8	1.2 – 2.6	2.9	2.2 – 3.9	4.3	3.4 – 5.5	2.2	1.6 – 3.1
**2019**	2.3	1.6 – 3.3	2.4	1.7 – 3.4	2.7	2.0 – 3.7	3.3	2.5 – 4.4	4.8	3.8 – 6.1	2.6	1.9 – 3.6	1.6	1.1 – 2.5	1.7	1.1 – 2.5	1.3	0.8 – 2.1	2.5	1.8 – 3.5	3.2	2.4 – 4.3	2.0	1.4 – 3.0
**2020**	1.5	1.0 – 2.3	2.3	1.7 – 3.2	2.5	1.8 – 3.4	3.6	2.8 – 4.7	6.8	5.6 – 8.1	3.2	2.4 – 4.2	1.9	1.4 – 2.8	1.6	1.1 – 2.3	1.3	0.9 – 2.1	2.7	2.0 – 3.6	4.4	3.5 – 5.5	2.4	1.7 – 3.3
**2021**	2.0	1.4 – 2.8	1.9	1.3 – 2.8	2.2	1.5 – 3.1	3.7	2.8 – 4.8	5.2	4.2 – 6.4	2.4	1.7 – 3.3	2.3	1.6 – 3.2	2.0	1.4 – 2.8	1.3	0.8 – 2.0	2.8	2.1 – 3.8	4.2	3.3 – 5.4	2.7	2.0 – 3.6
**2022**	2.3	1.6 – 3.2	2.8	2.1 – 3.8	2.1	1.5 – 3.0	3.8	2.9 – 4.9	6.1	4.9 – 7.4	3.0	2.2 – 4.0	3.3	2.5 – 4.3	2.2	1.5 – 3.1	1.7	1.1 – 2.5	3.2	2.4 – 4.2	4.9	3.9 – 6.1	2.4	1.7 – 3.4
**2023**	3.1	2.3 – 4.1	3.3	2.5 – 4.3	2.0	1.4 – 2.9	3.7	2.9 – 4.8	7.4	6.2 – 8.8	3.0	2.3 – 4.0	2.7	2.0 – 3.7	1.9	1.3 – 2.7	1.1	0.7 – 1.8	3.4	2.6 – 4.4	4.4	3.5 – 5.6	2.7	2.0 – 3.6

Note: CDS = Child Development Scale

Table S6. Annual valid sample sizes for the Index of Child Care Environment and Child Development Scale variables

Year	Sample size	Baseline ICCE(t), n (%)					Follow-up ICCE(t+1), n (%)				
		AR	HS	SST	SSU	Total ICCE	AR	HS	SST	SSU	Total ICCE
**1999**	2317	1381 (59.6)	1264 (54.6)	1500 (64.7)	1080 (46.6)	775 (33.4)	1385 (59.8)	1180 (50.9)	1394 (60.2)	1036 (44.7)	793 (34.2)
**2000**	2411	1406 (58.3)	1209 (50.1)	1416 (58.7)	1052 (43.6)	810 (33.6)	1496 (62.0)	1238 (51.3)	1498 (62.1)	1398 (58.0)	1081 (44.8)
**2001**	2303	1443 (62.7)	1181 (51.3)	1440 (62.5)	1332 (57.8)	1005 (43.6)	1655 (71.9)	1375 (59.7)	1654 (71.8)	1561 (67.8)	1235 (53.6)
**2002**	2311	1664 (72.0)	1422 (61.5)	1665 (72.0)	1596 (69.1)	1246 (53.9)	1657 (71.7)	1412 (61.1)	1664 (72.0)	1554 (67.2)	1256 (54.3)
**2003**	2191	1611 (73.5)	1348 (61.5)	1620 (73.9)	1498 (68.4)	1198 (54.7)	1517 (69.2)	1275 (58.2)	1536 (70.1)	1402 (64.0)	1151 (52.5)
**2004**	1579	1194 (75.6)	1032 (65.4)	1215 (76.9)	1135 (71.9)	909 (57.6)	1124 (71.2)	941 (59.6)	1124 (71.2)	1070 (67.8)	853 (54.0)
**2005**	1361	1028 (75.5)	868 (63.8)	1024 (75.2)	989 (72.7)	778 (57.2)	989 (72.7)	832 (61.1)	994 (73.0)	933 (68.6)	748 (55.0)
**2006**	1511	1108 (73.3)	929 (61.5)	1110 (73.5)	1060 (70.2)	828 (54.8)	1088 (72.0)	934 (61.8)	1104 (73.1)	1055 (69.8)	854 (56.5)
**2007**	1353	926 (68.4)	787 (58.2)	928 (68.6)	883 (65.3)	715 (52.8)	903 (66.7)	794 (58.7)	931 (68.8)	860 (63.6)	710 (52.5)
**2008**	1221	914 (74.9)	811 (66.4)	932 (76.3)	877 (71.8)	718 (58.8)	866 (70.9)	760 (62.2)	881 (72.2)	834 (68.3)	693 (56.8)
**2009**	1027	632 (61.5)	560 (54.5)	644 (62.7)	613 (59.7)	492 (47.9)	427 (41.6)	359 (35.0)	431 (42.0)	408 (39.7)	319 (31.1)
**2010**	435	109 (25.1)	91 (20.9)	114 (26.2)	105 (24.1)	80 (18.4)	148 (34.0)	123 (28.3)	147 (33.8)	140 (32.2)	112 (25.7)
**2011**	632	256 (40.5)	213 (33.7)	258 (40.8)	249 (39.4)	199 (31.5)	294 (46.5)	268 (42.4)	288 (45.6)	272 (43.0)	254 (40.2)
**2012**	1356	747 (55.1)	709 (52.3)	740 (54.6)	721 (53.2)	682 (50.3)	980 (72.3)	872 (64.3)	974 (71.8)	943 (69.5)	834 (61.5)
**2013**	1670	959 (57.4)	834 (49.9)	944 (56.5)	915 (54.8)	793 (47.5)	1024 (61.3)	1030 (61.7)	1067 (63.9)	1033 (61.9)	922 (55.2)
**2014**	1709	1112 (65.1)	1108 (64.8)	1153 (67.5)	1114 (65.2)	1014 (59.3)	1031 (60.3)	1046 (61.2)	1081 (63.3)	1057 (61.8)	973 (56.9)
**2015**	1690	919 (54.4)	926 (54.8)	961 (56.9)	932 (55.1)	850 (50.3)	1021 (60.4)	1049 (62.1)	1097 (64.9)	1070 (63.3)	959 (56.7)
**2016**	1802	1070 (59.4)	1075 (59.7)	1116 (61.9)	1096 (60.8)	1009 (56.0)	1258 (69.8)	1220 (67.7)	1251 (69.4)	1248 (69.3)	1186 (65.8)
**2017**	1977	1354 (68.5)	1317 (66.6)	1353 (68.4)	1346 (68.1)	1287 (65.1)	1360 (68.8)	1331 (67.3)	1350 (68.3)	1333 (67.4)	1305 (66.0)
**2018**	1680	1162 (69.2)	1137 (67.7)	1152 (68.6)	1141 (67.9)	1117 (66.5)	1079 (64.2)	1051 (62.6)	1075 (64.0)	1062 (63.2)	1035 (61.6)
**2019**	1467	1016 (69.3)	993 (67.7)	1013 (69.1)	1004 (68.4)	986 (67.2)	885 (60.3)	869 (59.2)	887 (60.5)	871 (59.4)	859 (58.6)
**2020**	1699	1003 (59.0)	991 (58.3)	1003 (59.0)	992 (58.4)	980 (57.7)	911 (53.6)	906 (53.3)	912 (53.7)	901 (53.0)	896 (52.7)
**2021**	1676	946 (56.4)	932 (55.6)	946 (56.4)	929 (55.4)	923 (55.1)	921 (55.0)	912 (54.4)	923 (55.1)	914 (54.5)	902 (53.8)
**2022**	1593	849 (53.3)	837 (52.5)	849 (53.3)	843 (52.9)	828 (52.0)	745 (46.8)	736 (46.2)	743 (46.6)	737 (46.3)	716 (44.9)
**2023**	1656	899 (54.3)	886 (53.5)	893 (53.9)	894 (54.0)	868 (52.4)	725 (43.8)	723 (43.7)	726 (43.8)	726 (43.8)	715 (43.2)

Note: AR = Avoidance Restriction; HS = Human Stimulation; SST = Social Stimulation; SSU = Social Support; ICCE, Index of Child Care Environment; CDS, Child Development Scale.

Table S7. Annual valid sample sizes for the Child Development Scale variables

Year	Sample size	Baseline CDS(t), n(%)						Follow-up CDS(t+1), n(%)					
		Gross motor	Fine motor	Social competence	Communication	Vocabulary	Intelligence	Gross motor	Fine motor	Social competence	Communication	Vocabulary	Intelligence
**1999**	2317	2083 (89.9)	2083 (89.9)	2097 (90.5)	2088 (90.1)	2055 (88.7)	2084 (89.9)	2211 (95.4)	2213 (95.5)	2218 (95.7)	2210 (95.4)	2187 (94.4)	2203 (95.1)
**2000**	2411	2263 (93.9)	2264 (93.9)	2270 (94.2)	2264 (93.9)	2229 (92.5)	2255 (93.5)	2270 (94.2)	2269 (94.1)	2279 (94.5)	2271 (94.2)	2249 (93.3)	2263 (93.9)
**2001**	2303	2190 (95.1)	2192 (95.2)	2199 (95.5)	2193 (95.2)	2164 (94.0)	2179 (94.6)	2189 (95.0)	2188 (95.0)	2196 (95.4)	2195 (95.3)	2173 (94.4)	2179 (94.6)
**2002**	2311	2210 (95.6)	2206 (95.5)	2208 (95.5)	2215 (95.8)	2187 (94.6)	2197 (95.1)	2228 (96.4)	2229 (96.5)	2236 (96.8)	2218 (96.0)	2223 (96.2)	2215 (95.8)
**2003**	2191	2135 (97.4)	2131 (97.3)	2143 (97.8)	2137 (97.5)	2120 (96.8)	2124 (96.9)	2135 (97.4)	2139 (97.6)	2141 (97.7)	2131 (97.3)	2130 (97.2)	2136 (97.5)
**2004**	1579	1558 (98.7)	1555 (98.5)	1558 (98.7)	1550 (98.2)	1542 (97.7)	1547 (98.0)	1559 (98.7)	1561 (98.9)	1562 (98.9)	1560 (98.8)	1554 (98.4)	1556 (98.5)
**2005**	1361	1347 (99.0)	1347 (99.0)	1348 (99.0)	1344 (98.8)	1341 (98.5)	1346 (98.9)	1337 (98.2)	1340 (98.5)	1339 (98.4)	1338 (98.3)	1334 (98.0)	1333 (97.9)
**2006**	1511	1487 (98.4)	1476 (97.7)	1477 (97.7)	1478 (97.8)	1469 (97.2)	1474 (97.6)	1494 (98.9)	1493 (98.8)	1494 (98.9)	1492 (98.7)	1493 (98.8)	1490 (98.6)
**2007**	1353	1298 (95.9)	1297 (95.9)	1297 (95.9)	1293 (95.6)	1293 (95.6)	1296 (95.8)	1318 (97.4)	1315 (97.2)	1316 (97.3)	1319 (97.5)	1310 (96.8)	1313 (97.0)
**2008**	1221	1204 (98.6)	1200 (98.3)	1203 (98.5)	1201 (98.4)	1185 (97.1)	1199 (98.2)	1207 (98.9)	1208 (98.9)	1208 (98.9)	1207 (98.9)	1200 (98.3)	1206 (98.8)
**2009**	1027	1010 (98.3)	1005 (97.9)	1009 (98.2)	1009 (98.2)	993 (96.7)	1004 (97.8)	1009 (98.2)	1009 (98.2)	1015 (98.8)	1011 (98.4)	999 (97.3)	1008 (98.1)
**2010**	435	429 (98.6)	427 (98.2)	432 (99.3)	432 (99.3)	427 (98.2)	429 (98.6)	411 (94.5)	334 (76.8)	335 (77.0)	332 (76.3)	334 (76.8)	332 (76.3)
**2011**	632	624 (98.7)	540 (85.4)	540 (85.4)	541 (85.6)	538 (85.1)	540 (85.4)	573 (90.7)	606 (95.9)	606 (95.9)	606 (95.9)	606 (95.9)	605 (95.7)
**2012**	1356	1300 (95.9)	1311 (96.7)	1311 (96.7)	1312 (96.8)	1309 (96.5)	1306 (96.3)	1264 (93.2)	1260 (92.9)	1252 (92.3)	1258 (92.8)	1262 (93.1)	1261 (93.0)
**2013**	1670	1593 (95.4)	1590 (95.2)	1580 (94.6)	1585 (94.9)	1589 (95.1)	1588 (95.1)	1595 (95.5)	1599 (95.7)	1606 (96.2)	1602 (95.9)	1595 (95.5)	1593 (95.4)
**2014**	1709	1510 (88.4)	1509 (88.3)	1549 (90.6)	1547 (90.5)	1516 (88.7)	1524 (89.2)	1569 (91.8)	1569 (91.8)	1569 (91.8)	1569 (91.8)	1567 (91.7)	1567 (91.7)
**2015**	1690	1628 (96.3)	1627 (96.3)	1627 (96.3)	1627 (96.3)	1625 (96.2)	1625 (96.2)	1533 (90.7)	1536 (90.9)	1534 (90.8)	1533 (90.7)	1532 (90.7)	1528 (90.4)
**2016**	1802	1641 (91.1)	1643 (91.2)	1640 (91.0)	1638 (90.9)	1637 (90.8)	1632 (90.6)	1706 (94.7)	1703 (94.5)	1702 (94.5)	1700 (94.3)	1702 (94.5)	1701 (94.4)
**2017**	1977	1887 (95.4)	1884 (95.3)	1884 (95.3)	1881 (95.1)	1880 (95.1)	1878 (95.0)	1885 (95.3)	1887 (95.4)	1888 (95.5)	1886 (95.4)	1884 (95.3)	1883 (95.2)
**2018**	1680	1610 (95.8)	1611 (95.9)	1611 (95.9)	1610 (95.8)	1608 (95.7)	1608 (95.7)	1637 (97.4)	1635 (97.3)	1637 (97.4)	1636 (97.4)	1637 (97.4)	1636 (97.4)
**2019**	1467	1435 (97.8)	1441 (98.2)	1440 (98.2)	1440 (98.2)	1440 (98.2)	1440 (98.2)	1424 (97.1)	1425 (97.1)	1423 (97.0)	1422 (96.9)	1421 (96.9)	1420 (96.8)
**2020**	1699	1644 (96.8)	1646 (96.9)	1644 (96.8)	1644 (96.8)	1642 (96.6)	1639 (96.5)	1648 (97.0)	1646 (96.9)	1646 (96.9)	1642 (96.6)	1641 (96.6)	1639 (96.5)
**2021**	1676	1598 (95.3)	1598 (95.3)	1601 (95.5)	1600 (95.5)	1602 (95.6)	1599 (95.4)	1571 (93.7)	1568 (93.6)	1569 (93.6)	1569 (93.6)	1564 (93.3)	1568 (93.6)
**2022**	1593	1527 (95.9)	1525 (95.7)	1526 (95.8)	1528 (95.9)	1518 (95.3)	1523 (95.6)	1568 (98.4)	1567 (98.4)	1569 (98.5)	1569 (98.5)	1567 (98.4)	1565 (98.2)
**2023**	1656	1604 (96.9)	1603 (96.8)	1590 (96.0)	1588 (95.9)	1588 (95.9)	1585 (95.7)	1602 (96.7)	1600 (96.6)	1602 (96.7)	1601 (96.7)	1599 (96.6)	1599 (96.6)

ICCE, Index of Child Care Environment; CDS, Child Development Scale.

Table S8. Cluster-Robust Sensitivity Analysis of the Main Associations

Outcome	ICCE	Original model						Child-cluster robust				Center-cluster robust			
		β	SE	OR	LCL	UCL	*p*	SE	LCL	UCL	*p*	SE	LCL	UCL	*p*
Gross motor	Avoidance restriction	0.057	0.100	1.059	0.871	1.288	0.566	0.098	0.874	1.284	0.560	0.142	0.802	1.399	0.687
	Human stimulation	-0.176	0.080	0.838	0.716	0.981	**0.028**	0.081	0.715	0.983	**0.029**	0.073	0.726	0.968	**0.016**
	Social stimulation	-0.141	0.072	0.869	0.754	1.001	0.052	0.074	0.751	1.005	0.059	0.092	0.726	1.040	0.126
	Social support	-0.111	0.093	0.895	0.746	1.073	0.230	0.097	0.739	1.083	0.252	0.086	0.756	1.059	0.196
	Total ICCE score	-0.094	0.038	0.911	0.845	0.982	**0.015**	0.037	0.846	0.980	**0.012**	0.049	0.827	1.003	0.058
Fine motor	Avoidance restriction	-0.266	0.073	0.766	0.665	0.884	**<0.001**	0.074	0.664	0.885	**<0.001**	0.088	0.645	0.911	**0.003**
	Human stimulation	-0.152	0.065	0.859	0.757	0.975	**0.019**	0.062	0.761	0.970	**0.014**	0.070	0.748	0.986	**0.031**
	Social stimulation	-0.046	0.059	0.955	0.851	1.072	0.434	0.062	0.846	1.078	0.458	0.073	0.827	1.102	0.530
	Social support	-0.141	0.070	0.868	0.757	0.995	**0.043**	0.075	0.749	1.007	0.061	0.065	0.764	0.987	**0.031**
	Total ICCE score	-0.119	0.030	0.888	0.838	0.941	**<0.001**	0.030	0.837	0.942	**<0.001**	0.034	0.830	0.950	**0.001**
Social competence	Avoidance restriction	-0.254	0.075	0.776	0.670	0.898	**0.001**	0.074	0.671	0.897	**0.001**	0.115	0.619	0.972	**0.028**
	Human stimulation	-0.113	0.069	0.893	0.780	1.023	0.102	0.069	0.780	1.022	0.100	0.082	0.760	1.049	0.167
	Social stimulation	-0.088	0.060	0.916	0.815	1.029	0.140	0.063	0.810	1.036	0.161	0.082	0.779	1.076	0.285
	Social support	-0.149	0.072	0.862	0.748	0.992	**0.039**	0.075	0.743	0.999	**0.049**	0.063	0.762	0.975	**0.018**
	Total ICCE score	-0.114	0.030	0.892	0.840	0.947	**<0.001**	0.030	0.841	0.946	**<0.001**	0.038	0.827	0.962	**0.003**
Communication	Avoidance restriction	-0.223	0.062	0.800	0.708	0.904	**<0.001**	0.063	0.707	0.905	**<0.001**	0.084	0.679	0.942	**0.008**
	Human stimulation	-0.154	0.056	0.858	0.768	0.957	**0.006**	0.056	0.769	0.956	**0.006**	0.073	0.744	0.989	**0.034**
	Social stimulation	-0.154	0.049	0.858	0.779	0.944	**0.002**	0.051	0.776	0.948	**0.003**	0.059	0.764	0.963	**0.009**
	Social support	-0.182	0.059	0.833	0.742	0.935	**0.002**	0.059	0.742	0.936	**0.002**	0.047	0.760	0.913	**<0.001**
	Total ICCE score	-0.122	0.025	0.886	0.843	0.930	**<0.001**	0.024	0.844	0.929	**<0.001**	0.029	0.836	0.938	**<0.001**
Vocabulary	Avoidance restriction	-0.187	0.054	0.830	0.747	0.922	**<0.001**	0.053	0.748	0.920	**<0.001**	0.078	0.712	0.967	**0.017**
	Human stimulation	-0.221	0.045	0.802	0.734	0.876	**<0.001**	0.046	0.733	0.877	**<0.001**	0.066	0.704	0.912	**0.001**
	Social stimulation	-0.071	0.042	0.931	0.858	1.011	0.090	0.044	0.855	1.015	0.104	0.062	0.824	1.053	0.255
	Social support	-0.199	0.049	0.819	0.744	0.903	**<0.001**	0.049	0.744	0.902	**<0.001**	0.041	0.755	0.889	**<0.001**
	Total ICCE score	-0.118	0.021	0.889	0.853	0.927	**<0.001**	0.021	0.853	0.926	**<0.001**	0.030	0.839	0.942	**<0.001**
Intelligence	Avoidance restriction	-0.240	0.061	0.786	0.698	0.886	**<0.001**	0.061	0.698	0.886	**<0.001**	0.092	0.656	0.942	**0.009**
	Human stimulation	-0.149	0.054	0.862	0.775	0.957	**0.006**	0.053	0.777	0.955	**0.005**	0.067	0.755	0.983	**0.027**

Social stimulation	-0.058	0.049	0.943	0.857	1.038	0.232	0.050	0.855	1.041	0.247	0.079	0.808	1.101	0.459
Social support	-0.144	0.058	0.866	0.772	0.971	**0.014**	0.059	0.771	0.973	**0.016**	0.044	0.794	0.945	**0.001**
Total ICCE score	-0.120	0.024	0.887	0.846	0.931	**<0.001**	0.024	0.846	0.930	**<0.001**	0.035	0.828	0.951	**0.001**

Table S9. Cluster-Robust Sensitivity Analysis of the Calendar-Year Interaction Associations

**Outcome**	**ICCE**	**Original Interaction model （ICCE × year）**						**Child-cluster robust**				**Center-cluster robust**			
		β	**SE**	**OR**	**LCL**	**UCL**	***p***	**SE**	**LCL**	**UCL**	***p***	**SE**	**LCL**	**UCL**	**p**
Gross motor	Avoidance restriction	0.001	0.013	1.000	0.974	1.027	0.989	0.014	0.973	1.027	0.989	0.015	0.970	1.031	0.990
	Human stimulation	-0.023	0.011	0.977	0.957	0.997	**0.028**	0.012	0.955	0.999	**0.043**	0.013	0.952	1.002	0.074
	Social stimulation	-0.036	0.009	0.965	0.947	0.982	**<0.001**	0.009	0.947	0.983	**<0.001**	0.008	0.950	0.979	**<0.001**
	Social support	0.009	0.012	1.010	0.987	1.033	0.415	0.012	0.986	1.034	0.431	0.016	0.978	1.042	0.558
	Total ICCE score	-0.016	0.005	0.984	0.974	0.994	**0.003**	0.005	0.975	0.994	**0.002**	0.006	0.973	0.996	**0.007**
Fine motor	Avoidance restriction	-0.005	0.011	0.995	0.974	1.017	0.659	0.010	0.976	1.014	0.615	0.009	0.978	1.012	0.577
	Human stimulation	-0.019	0.008	0.981	0.965	0.997	**0.021**	0.007	0.967	0.995	**0.008**	0.007	0.967	0.994	**0.006**
	Social stimulation	-0.028	0.008	0.972	0.957	0.987	**<0.001**	0.008	0.956	0.988	**0.001**	0.007	0.958	0.986	**<0.001**
	Social support	<0.001	0.010	1.000	0.981	1.019	0.975	0.009	0.982	1.018	0.973	0.013	0.975	1.025	0.981
	Total ICCE score	-0.010	0.004	0.990	0.982	0.998	**0.012**	0.004	0.982	0.998	**0.012**	0.005	0.981	0.999	**0.022**
Social competence	Avoidance restriction	0.007	0.012	1.007	0.983	1.031	0.583	0.010	0.988	1.026	0.489	0.011	0.985	1.029	0.540
	Human stimulation	-0.024	0.009	0.976	0.959	0.994	**0.008**	0.009	0.960	0.993	**0.006**	0.011	0.956	0.997	**0.025**
	Social stimulation	-0.033	0.008	0.967	0.952	0.983	**<0.001**	0.010	0.949	0.986	**0.001**	0.010	0.949	0.986	**0.001**
	Social support	-0.005	0.010	0.995	0.974	1.015	0.601	0.009	0.976	1.013	0.561	0.012	0.972	1.018	0.644
	Total ICCE score	-0.012	0.004	0.988	0.980	0.997	**0.008**	0.005	0.980	0.997	**0.010**	0.006	0.978	0.999	**0.037**
Communication	Avoidance restriction	<0.001	0.009	1.000	0.981	1.018	0.963	0.008	0.983	1.016	0.959	0.007	0.987	1.013	0.948
	Human stimulation	-0.023	0.007	0.977	0.964	0.991	**0.001**	0.007	0.964	0.990	**0.001**	0.007	0.964	0.991	**0.001**
	Social stimulation	-0.028	0.007	0.972	0.960	0.985	**<0.001**	0.006	0.960	0.985	**<0.001**	0.007	0.959	0.985	**<0.001**
	Social support	-0.008	0.008	0.992	0.976	1.007	0.290	0.007	0.977	1.006	0.256	0.007	0.978	1.005	0.216
	Total ICCE score	-0.014	0.003	0.987	0.980	0.993	**<0.001**	0.003	0.980	0.993	**<0.001**	0.003	0.981	0.993	**<0.001**
Vocabulary	Avoidance restriction	0.002	0.008	1.002	0.986	1.017	0.832	0.007	0.988	1.016	0.816	0.009	0.984	1.019	0.850
	Human stimulation	-0.024	0.006	0.976	0.965	0.988	**<0.001**	0.006	0.965	0.988	**<0.001**	0.006	0.966	0.987	**<0.001**
	Social stimulation	-0.023	0.006	0.977	0.967	0.988	**<0.001**	0.006	0.966	0.988	**<0.001**	0.006	0.966	0.989	**<0.001**

	Social support	-0.014	0.007	0.986	0.973	0.999	**0.031**	0.006	0.974	0.998	**0.025**	0.006	0.975	0.997	**0.015**
	Total ICCE score	-0.013	0.003	0.987	0.981	0.992	**<0.001**	0.003	0.981	0.992	**<0.001**	0.003	0.982	0.992	**<0.001**
Intelligence	Avoidance restriction	-0.002	0.010	0.998	0.980	1.017	0.847	0.008	0.982	1.014	0.823	0.007	0.985	1.012	0.791
	Human stimulation	-0.031	0.007	0.970	0.956	0.983	**<0.001**	0.006	0.957	0.982	**<0.001**	0.006	0.958	0.982	**<0.001**
	Social stimulation	-0.031	0.007	0.970	0.957	0.983	**<0.001**	0.007	0.957	0.983	**<0.001**	0.009	0.952	0.988	**0.001**
	Social support	-0.019	0.008	0.981	0.966	0.996	**0.015**	0.008	0.966	0.996	**0.013**	0.008	0.966	0.995	**0.010**
	Total ICCE score	-0.017	0.003	0.983	0.977	0.990	**<0.001**	0.003	0.977	0.990	**<0.001**	0.004	0.976	0.991	**<0.001**
